# Neuropathological Characterization of a Dravet Syndrome Knock-In Mouse Model Useful for Investigating Cannabinoid Treatments

**DOI:** 10.3389/fnmol.2020.602801

**Published:** 2021-01-29

**Authors:** Valentina Satta, Cristina Alonso, Paula Díez, Soraya Martín-Suárez, Marta Rubio, Juan M. Encinas, Javier Fernández-Ruiz, Onintza Sagredo

**Affiliations:** ^1^Instituto Universitario de Investigación en Neuroquímica, Departamento de Bioquímica y Biología Molecular, Facultad de Medicina, Universidad Complutense, Madrid, Spain; ^2^Centro de Investigación Biomédica en Red de Enfermedades Neurodegenerativas (CIBERNED), Madrid, Spain; ^3^Instituto Ramón y Cajal de Investigación Sanitaria (IRYCIS), Madrid, Spain; ^4^The NSC Cell and Neurogenesis Laboratory, Achucarro Basque Center for Neuroscience, Leioa, Spain; ^5^The University of the Basque Country (UPV/EHU), Leioa, Spain; ^6^IKERBASQUE, The Basque Foundation for Science, Bilbao, Spain

**Keywords:** Dravet syndrome, infantile epileptic refractory syndromes, Syn-Cre/Scn1a^WT/A1783V^ mice, neuropathological characterization, cannabinoids, endocannabinoid signaling, inflammation, neurogenesis

## Abstract

Dravet syndrome (DS) is an epileptic syndrome caused by mutations in the *Scn1a* gene encoding the α1 subunit of the sodium channel Nav1.1, which is associated with febrile seizures that progress to severe tonic-clonic seizures and associated comorbidities. Treatment with cannabidiol has been approved to reduce seizures in DS, but it may also be active against these comorbidities. The aim of this study was to validate a new mouse model of DS having lower mortality than previous models, which may serve to further evaluate therapies for the long-term comorbidities. This new model consists of heterozygous conditional knock-in mice carrying a missense mutation (A1783V) in *Scn1a* gene expressed exclusively in neurons of the CNS (Syn-Cre/Scn1a^WT/A1783V^). These mice have been used here to determine the extent and persistence of the behavioral deterioration in different postnatal days (PND), as well as to investigate the alterations that the disease produces in the endocannabinoid system and the contribution of inflammatory events and impaired neurogenesis in the pathology. Syn-Cre/Scn1a^WT/A1783V^ mice showed a strong reduction in hindlimb grasp reflex at PND10, whereas at PND25, they presented spontaneous convulsions and a greater susceptibility to pentylenetetrazole-induced seizures, marked hyperactivity, deficient spatial working memory, lower levels of anxiety, and altered social interaction behavior. These differences disappeared at PND40 and PND60, except the changes in social interaction and anxiety. The analysis of CNS structures associated with these behavioral alterations revealed an elevated glial reactivity in the prefrontal cortex and the dentate gyrus. This was associated in the dentate gyrus with a greater cell proliferation detected with Ki67 immunostaining, whereas double-labeling analyses identified that proliferating cells were GFAP-positive suggesting failed neurogenesis but astrocyte proliferation. The analysis of the endocannabinoid system of Syn-Cre/Scn1a^WT/A1783V^ mice confirmed reductions in CB_1_ receptors and MAGL and FAAH enzymes, mainly in the cerebellum but also in other areas, whereas CB_2_ receptors became upregulated in the hippocampus. In conclusion, Syn-Cre/Scn1a^WT/A1783V^ mice showed seizuring susceptibility and several comorbidities (hyperactivity, memory impairment, less anxiety, and altered social behavior), which exhibited a pattern of age expression similar to DS patients. Syn-Cre/Scn1a^WT/A1783V^ mice also exhibited greater glial reactivity and a reactive response in the neurogenic niche, and regional changes in the status of the endocannabinoid signaling, events that could contribute in behavioral impairment.

## Introduction

Dravet syndrome (DS), also known as severe myoclonic epilepsy of infancy, was described in 1978 by Dravet (1978). DS is a rare genetic pediatric epileptic encephalopathy whose incidence is estimated around 1:20,000 subjects, representing 1.4% of children with epilepsy (Skluzacek et al., 2011). DS typically presents around 5 to 8 months of age with febrile seizures that progress to severe partial or generalized tonic-clonic seizures, myoclonic seizures, atypical absences, and focal seizures, as well as episodes of status epilepticus (in 80% of cases during the first year of life) (Skluzacek et al., 2011). DS is often accompanied by important comorbidities such as cognitive impairment and intellectual disability, behavioral disturbances with hyperactivity, attention deficits, and sometimes autistic traits, having also a higher incidence of sudden unexpected death (Skluzacek et al., 2011; Guerrini, 2012). Seizures tend to become less frequent during adolescence and, in some cases, disappear in adulthood, despite long-term cognitive, emotional, speech, and motor deficits frequently remaining in patients (Darra et al., 2019). In ~80% of patients, DS is caused by haploinsufficiency derived from *de novo* mutations in *Scn1a*, the gene encoding the α-subunit of the voltage-gated sodium channel Nav1.1, mutations that lead to a loss of function in this channel (Bender et al., 2012; Guerrini, 2012). This loss of function in Nav1.1 causes a marked reduction in the sodium current and an impairment in the firing of many types of GABAergic interneurons, in particular the fast-spiking parvalbumin-positive interneurons, where Nav1.1. is primarily located (Oakley et al., 2001; Bender et al., 2012). This results in an imbalance between excitation and inhibition, likely contributing to hyperexcitability and seizures (Cheah et al., 2012) and alterations in the normal function of neural networks known to be critical for the cognitive processes (Bender et al., 2012).

Given the genetic origin of this disease, numerous knock-out, knock-in, and transgenic models (most of them in mice) have been developed (Oakley et al., 2001; Yu et al., 2006; Ogiwara et al., 2007; Tang et al., 2009; Martin et al., 2010; Cheah et al., 2012; Dutton et al., 2013; Miller et al., 2014; Rubinstein et al., 2015; Tsai et al., 2015; Griffin et al., 2018), so that they may be used to better understand the molecular and cellular mechanisms underlying the physiopathology of DS, as well as to investigate novel treatments. For example, the use of these models has primarily served to investigate the mechanism of epileptogenesis in DS, which is hypothesized, as mentioned above, to be a reduction in GABA-dependent inhibitory transmission leading to brain hyperexcitability and seizures (Ogiwara et al., 2007; Tai et al., 2014). The most common murine models for DS are based on total or partial deletions of *Scn1a* gene or expression of specific human point mutations found in this gene, always under different promoters and allowing total or conditional (using Cre-*loxP* strategies) gene manipulations (Oakley et al., 2001; Yu et al., 2006; Ogiwara et al., 2007; Tang et al., 2009; Martin et al., 2010; Cheah et al., 2012; Dutton et al., 2013; Miller et al., 2014; Rubinstein et al., 2015; Tsai et al., 2015; Griffin et al., 2018). These models, in general, recapitulate the different characteristics of this pathology, mainly the seizuring activity, but with different timings and extents, then serving for different experimental purposes (reviewed in Griffin et al., 2018). For example, the models based on homozygous deletion of the *Scn1a* gene present premature mortality (before 2 weeks after birth, Ogiwara et al., 2007), whereas the heterozygous deletion leads to haploinsufficiency generating spontaneous seizures, delayed mortality (from 3 to 12 weeks of age having only 20% of long-term survival), and invalidating comorbidities (Yu et al., 2006). Models based on the conditional deletion (or expression of *Scn1a* mutations) in specific cell populations, for example, in hippocampal neurons (Stein et al., 2019), forebrain interneurons (Cheah et al., 2012; Han et al., 2012), or parvalbumin-positive interneurons (Tatsukawa et al., 2018), have also been developed and served to investigate the relation of these cell populations with DS, and more specifically with some comorbidities (e.g., hyperactivity, altered anxiety-like behavior, excessive stereotyped behaviors, deficits in social interaction behavior, spatial learning, and memory impairment) frequently associated with the disease.

The fact that DS pathogenesis still remains poorly understood explains the limited therapeutic development for this disease to date. The antiepileptic drugs used as first-line therapy are valproate, topiramate, and clobazam, but the response to these drugs is often inadequate (patients are usually drug-resistant), and, although it improves when stiripentol is used as an adjuvant (Frampton, 2019), they have limited efficacy and produce adverse effects, so there is an urgent need of new therapies for DS (Bialer et al., 2018; Brigo et al., 2018). Data generated along the last 6 years suggest that a promising therapy for DS may be based on the use of certain cannabinoids, e.g., cannabidiol (CBD) (Franco and Perucca, 2019; Morano et al., 2020), which not only target the so-called endocannabinoid system but also other elements outside this signaling system (Gray and Whalley, 2020). This has been demonstrated in a series of clinical studies conducted in patients affected by DS, Lennox-Gastaut syndrome, or similar pathologies, using CBD, formulated as Epidiolex®, and concentrating on its activity on seizures (Porter and Jacobson, 2013; Devinsky et al., 2016, 2018; Nabbout and Thiele, 2020). These studies demonstrated that CBD was well tolerated and reduced seizure frequency and intensity in these patients (Porter and Jacobson, 2013; Devinsky et al., 2016, 2018; Nabbout and Thiele, 2020), which led to the recent approval of Epidiolex® by some regulatory agencies as an anticonvulsant agent for these treatment-resistant infantile epilepsies^1^^,^^2^, being currently under investigation for tuberous sclerosis complex (Hess et al., 2016). However, beyond the anticonvulsant properties of Epidiolex®, CBD, given its well-demonstrated anti-inflammatory, antioxidant, and cytoprotective properties (Fernández-Ruiz et al., 2013), may also be beneficial against important comorbidities associated with the disease, such as the long-term cognitive, motor, emotional, and speech deterioration, a fact already investigated in a couple of recent studies conducted in experimental models of DS (Kaplan et al., 2017; Patra et al., 2020) and also after hippocampal kainate lesions in juvenile rats (Friedman and Wongvravit, 2018). This possible long-term efficacy of CBD might be related to a correction of a possible dysregulation of the endocannabinoid signaling system [which would contain potential pharmacological targets for CBD (Fernández-Ruiz et al., 2013)] occurring in the CNS, as has been found in other neurological disorders (Cristino et al., 2020). Such dysregulation in the endocannabinoid signaling was recently proposed also for DS based on studies carried out in lymphocytes from DS patients (Rubio et al., 2016), but the evidence is indirect and would need to be confirmed in animal models of DS. The possibility of a dysregulation in the endocannabinoid system was not investigated in the two studies that explored the efficacy of CBD against DS-associated comorbidities (Kaplan et al., 2017; Patra et al., 2020), so to explore this possibility has been the final objective of our present study. These two previous studies (Kaplan et al., 2017; Patra et al., 2020) were carried out in models of heterozygous deletion of *Scn1a* gene, which, as mentioned above, recapitulate DS features because of the loss of function of DS mutations in this gene. However, these mice exhibited a high premature mortality (Yu et al., 2006), which makes difficult, in general, to progress in the study of novel therapies for the treatment of long-term comorbidities, and, in the case of cannabinoids, to investigate the contribution of possible endocannabinoid dysregulations in the DS pathogenesis and/or in the therapy efficacy. This was an important consideration for the design of our present study, which was conducted in a novel murine model of DS based on the expression of the A1783V mutation in the exon 26 of the *Scn1a* gene instead on the classic deletion of this gene. A1783V is one of the most frequent missense mutations described so far (Lossin, 2009) and, overexpressed in animal models, has been found to be associated with important motor and cognitive disturbances reminiscent of DS (Ricobaraza et al., 2019), so it may represent an ideal model for future studies on disease-modifying therapies in DS, including CBD and other cannabinoids. A similar model was developed recently by Ricobaraza et al. (2019), which resulted to be an excellent tool for the study of numerous pathogenic aspects of DS. However, this mouse model also presented high rates of mortality (>75% at 40 days of age), which may again limit its use for investigating therapies for long-term comorbidities. In these DS mice, the A1783V mutation is expressed in all body cells [using mice with Cre-recombinase (Cre) under the CMV promoter; see (Ricobaraza et al., 2019)]. Therefore, for our study, we crossed heterozygous conditional knock-in mice [B6(Cg)-Scn1atm1.1Dsf/J] carrying the missense A1783V mutation in *Scn1a* gene with mice expressing Cre linked to synapsin-1 promoter [CreB6.Cg-Tg(Syn1-cre)671Jxm/J], then resulting in the generation of mice with the A1783V mutation exclusively in CNS neurons (Syn-Cre/Scn1a^WT/A1783V^) and three different control mice (Scn1a^WT/WT^, Syn-Cre/Scn1a ^WT/WT^, and Scn1a ^WT/A1783V^). Such strategy should elevate mouse survival, facilitating the study of long-term therapies, as well as provide information on the specific role played by the mutant channel in the progression of the disease when expressed exclusively in CNS neurons. Therefore, the objectives of our study were first to characterize this new conditional knock-in mouse model of DS paying emphasis on its survival, its seizure susceptibility, and its responses in motor, cognitive, anxiety, and social interaction tests at different postnatal days (PND). Our second objective was to determine the contribution to these behavioral disturbances of inflammatory events occurring in specific CNS areas that control these behavioral processes, as well as the contribution of alterations in hippocampal neurogenesis which have been associated with cognitive impairment in epileptic conditions (Muro-García et al., 2019; Martín-Suárez et al., 2020). Both objectives were followed by the above-mentioned attempt to identify the alterations that the disease produces in the endocannabinoid system, which would contain the potential targets for any tentative cannabinoid-based therapy. As mentioned above, the final expectation is that these mice may be furtherly used to confirm the benefits of an anti-inflammatory therapy with CBD (or other cannabinoids) against the long-term deficits found in this disease, using, in this case, a model based on the conditional expression of A1783V mutation rather than the models generated by heterozygous deletion of *Scn1a* gene used in previous studies (Kaplan et al., 2017; Patra et al., 2020).

## Materials and Methods

### Animals, Genotyping, Experiments, and Sampling

We used conditional knock-in mutant mice (knock-in mutation A1783V in Nav1.1 protein) generated by Cre-*loxP* technology, in which the *Scn1a* gene is primarily mutated in neuronal cells. With this purpose, B6(Cg)-Scn1atm1.1Dsf/J mice (heterozygous for the transgene, JAX stock #026133) were crossed with Cre-recombinase linked to synapsin-1 promoter mice (CreB6.Cg-Tg(Syn1-cre)671Jxm/J; JAX stock #003966), both acquired from The Jackson Laboratory (Bar Harbor, ME, USA), breedings which gave rise to offspring in one of the four following experimental groups: the Syn-Cre/Scn1a^WT/A1783V^ mice, which bear the A1783V mutation in Nav1.1 protein exclusively in CNS neurons, showing the pathological phenotype, and their three different control mice, Scn1a^WT/WT^ (wild type not expressing Cre), Syn-Cre/Scn1a^WT/WT^ (wild type expressing Cre), and Scn1a^WT/A1783V^ (A1783V mutant not expressing Cre), which show, in general, a normal phenotype. Genotypes were verified by polymerase chain reaction (PCR) using genomic DNA from mouse tail biopsies. Genomic DNA was extracted and amplified using REDExtract-N-Amp Tissue PCR kit (Sigma-Aldrich, Madrid, Spain) and following manufacturer's instructions. Specific pairs of primers used are indicated in Table 1. PCR products were separated by electrophoresis in 2% agarose gels and visualized using GelRed. A representative PCR is shown in Supplementary File 1. During the experiments, mice were housed in a room with a controlled photoperiod (08:00–20:00 light) and temperature (22 ± 1°C), and with free access to food and water. All experiments were conducted according to the national and European guidelines (RD 53/2013 and directive 2010/63/EU, respectively), following the principles of the ARRIVE and 3R guidelines, and were approved by the Animal Welfare Committee of the Complutense University and the “Comunidad de Madrid” (ref. PROEX 033/17). All possible efforts were made to minimize animal pain and discomfort, as well as reduce the number of experimental subjects.

**Table 1:** Sequences of primers used for genotyping by PCR.

**Gene**	**Primers**
Scn1a	F: 5′-TAC TGG GAT CCA CCT CCA CT-3′
	R: 5′-TAG CTC CGC AAG AAA CAT CC-3′
Syn-Cre	F: 5′-CTC AGC GCT GCC TCA GTC T-3′
	R: 5′-GCA TCG ACC GGT AAT GCA-3′

The study was divided in two experiments. In the first one, Syn-Cre/Scn1a^WT/A1783V^ mice and their different controls (Scn1a^WT/WT^, Syn-Cre/Scn1a^WT/WT^, and Scn1a^WT/A1783V^) were analyzed at different PNDs in some behavioral tests (grasp reflexes, rotarod, computer-aided actimeter, Y-maze, elevated plus maze, and social interaction). The period investigated included from PND10 (only grasp reflexes) up to PND60 (all tests). In a second experiment, and given that PND25 was the age at which behavioral impairment was maximal, we generated Syn-Cre/Scn1a^WT/A1783V^ mice and controls (only Scn1a^WT/WT^, given that no differences were found among the three control groups in the first experiment), which were used at this age for two purposes. On one hand, we were interested in analyzing the seizure activity of Syn-Cre/Scn1a^WT/A1783V^ mice compared with their controls after being both acutely injected with the proconvulsant agent PTZ. On the other hand, an additional cohort of Syn-Cre/Scn1a^WT/A1783V^ and Scn1a^WT/WT^ mice was euthanized by rapid decapitation at PND25 and brains were rapidly removed and divided into the two hemispheres. Left hemispheres were fixed for 1 day at 4°C in fresh 4% paraformaldehyde prepared in 0.1 M phosphate-buffered saline (PBS), pH 7.4, then cryoprotected by immersion in a 30% sucrose solution for a further day, and finally stored at −80°C for immunohistochemical analysis. Right hemispheres were rapidly dissected in different brain areas of interest, such as prefrontal cortex, striatum, hippocampus, and cerebellum, which were frozen in 2-methylbutane cooled in dry ice and stored at −80°C until processing for western blot or qPCR analysis of different proteins of interest, including endocannabinoid elements.

### Behavioral Recording

The behavioral tests were performed to detect possible motor deficits (limb grasp reflexes, rotarod test, computer-aided actimeter), cognitive and emotional impairments (Y-maze and elevated-plus maze tests), and autistic-like traits (social interaction test) at some representative ages after birth: PND10 (only reflexes), PND25, PND40, and PND60 (all tests). Mice were transferred to the behavior room and acclimated for 1 h before testing to prevent that a novel environment can modify the behavioral response. Tests were carried out in the following order in two consecutive days: open-field and social interaction test were performed the day before reaching each age, followed by Y-maze and elevated plus-maze performed at each specific age. In behavioral assays, all animals were tested during the light cycle at the same time of the day (to 9:00 until 12:00 a.m.) in a room illuminated by a dim lamp (~50 lux), and each individual mouse was tested no more than twice per day. In all cases, the equipment was cleaned with 70% ethanol before and after testing of each mouse.

#### Analysis of Limb Grasp Reflexes

Forelimb and hindlimb grasp reflexes were measured as an index of motor development (Blaney et al., 2013). Mice were held on their back, and the fore- and hindlimbs were touched with a thin rod of 1 mm diameter. A score of 1 was assigned if the stimulation caused the fore- or hindlimb to grasp, whereas a score of 0 was assigned if no response was reached.

#### Vibrissae Response

Vibrissae response was measured to record the development of the tactile sensory system (Blaney et al., 2013). Mice were held by their tail and approached toward a flat surface. A score of 1 was assigned if mice raised their head to avoid contact with the flat surface due to vibrissae stimulation, whereas a score of 0 was assigned if no response was reached.

#### Computer-Aided Actimeter

Motor activity was analyzed in a computer-aided actimeter (Actitrack, Panlab, Barcelona, Spain) (Palomo-Garo et al., 2016). This apparatus consisted of a 45 × 45 cm area, with infrared beams all around, spaced 2.5 cm, coupled to a computerized control unit that analyzes the following parameters: (i) distance run in the actimeter (ambulation); (ii) frequency of vertical activity (rearing); (iii) resting time; (iv) mean velocity spent in ambulation; and (v) time spent in fast movements (>5 cm/s). Animals remained for a period of 40 min in the actimeter, and measurements were recorded during each 10 min (first 10 min was used only for animal acclimation).

#### Rotarod Test

Motor coordination was measured in a LE8200 device (Panlab, Barcelona, Spain) following a procedure previously described (Palomo-Garo et al., 2016). After a period of acclimation and training (first session: 0 rpm for 10 s; second and third sessions: 4 rpm for 10 s), animals were tested with an acceleration from 4 to 40 rpm over a period of 300 s. Mice were tested for 3 consecutive trials and the mean of the 3 trials was calculated.

#### Elevated Plus Maze

Animal anxiety was measured in the elevated plus maze that consisted of two opposite closed (30 × 5 × 15 cm) and open (30 × 5 cm) arms forming a plus-shaped maze. The structure was elevated to a height of 40 cm from the floor and all four arms were connected at right angle at a central area. At the beginning of a trial, each animal was placed in the intersection of the four arms (facing an open arm) and were left free to explore the maze for 5 min. During this test period, the following behavioral measures were recorded: (i) number of open and closed arm entries; and (ii) time spent in the open and closed arms. Closed-arm entries (counts/5 min) were considered indicators of locomotor activity, whereas open-arm exploration (% time spent in open arms and % open arm entries) were used as measures of anxiety (Walf and Frye, 2007).

#### Y-Maze Test

The Y-maze is a behavioral test used to measure the spatial working memory in mice based on the capacity of rodents to explore new environments. The apparatus is a polyvinyl plastic horizontal maze formed by 3 arms (40 × 12 × 3 cm) placed at 120° angles to each other and designated as A, B, and C. Each animal was placed in the center of the maze and was allowed to freely explore the three arms. The sequence (i.e., ABCCAB, etc.) and the number of arm entries were recorded during 8 min. The spontaneous alternation performance (SAP), a score of three consecutive different arm entries (ABC, CAB, or BCA), was analyzed. Memory impairments are also evaluated by the frequency of same arm returns (SAR) or alternate arm return (AAR). The alternation percentage was calculated according to the equation follows: % alternation = [(number of alternations) / (total arm entries)] × 100 (Joshi et al., 2014).

#### Social Interaction Test

This test is frequently used to determine social anxiety. It is conducted in an open-field arena (45 × 45 cm) in which each experimental animal was placed together with a novel unfamiliar congener in an environment and was allowed to freely explore him. The behavior was recorded for 20 min, and the total time spent by the experimental mouse in nonaggressive social interactions such as sniffing, following, or grooming the partner, was monitored (Silverman et al., 2010). Also, the number of active contacts with the partner was scored.

#### Analysis of PTZ-Induced Seizuring Activity

Seizuring activity was induced by administration of the proconvulsant PTZ, following a previous method (Alachkar et al., 2020). PTZ (Sigma-Aldrich, Madrid, Spain) was freshly dissolved in sterile saline (0.9% NaCl) to prepare a solution with a concentration of 10 mg/ml. The test was conducted on mice at PND25 which where individually placed in glass boxes and injected with PTZ (50 mg/kg, i.p.) and were then observed for a 30-min period. During this period, several behavioral parameters were recorded: (i) latency to myoclonic jerks, (ii) latency to generalized seizures, and (iii) total duration of generalized seizures.

### Real-Time qRT-PCR Analysis

Total RNA was extracted from prefrontal cortex, striatum, hippocampus, and cerebellum of right brain hemisphere samples using SurePrep^TM^ RNA/Protein Purification kit (Fisher BioReagents, FairLawn, NJ, USA). The total amount of RNA extracted was quantitated by spectrometry at 260 nm, and its purity was evaluated by the ratio between the absorbance values at 260 and 280 nm, whereas its integrity was confirmed in agarose gels. To prevent genomic DNA contamination, DNA was removed and single-stranded complementary DNA was synthesized from 0.2 μg of total RNA using a commercial kit (Rneasy Mini Quantitect Reverse Transcription, Qiagen, Izasa, Madrid, Spain). The reaction mixture was kept frozen at −80°C until enzymatic amplification. Quantitative real-time PCR assays were performed using Taq Man Gene Expression Assays (Applied Biosystems, Foster City, CA, USA) to quantify mRNA levels for CB_1_ receptor (ref. Mm00432621_s1), CB_2_ receptor (ref. Mm00438286_m1), GPR55 (ref. Mm03978245_m1), FAAH (ref. Mm00515684_m1), MAGL (ref. Mm00449274_m1), DAGL (ref. Mm00813830_m1), NAPE-PLD (ref. Mm00724596_m1), TNF-α (ref. Mm99999068_m1), and BDNF (ref. Mm01334042_m1), using GAPDH expression (ref. Mm99999915_g1) as an endogenous control gene for normalization. The PCR assay was performed using the 7300 Fast Real-Time PCR System (Applied Biosystems, Foster City, CA, USA), and the threshold cycle (Ct) was calculated by the instrument's software (7300 Fast System, Applied Biosystems, Foster City, CA, USA). Expression levels were calculated using the 2-ΔΔCt method, but, for presentation, data were transformed to the percentage over the mean obtained in the wild-type group.

### Western Blot

Frozen tissues were homogenized in ice-cold radioimmunoprecipitation assay (RIPA) buffer for protein extraction. Homogenates were centrifuged at 10,000 × *g* for 15 min at 4°C. Bio-Rad DC protein assay kit (Bio-Rad Laboratories, CA, USA) was used to quantify protein concentration, using bovine serum albumin (BSA) as the standard protein. Then, 15 μg of protein were boiled for 5 min in Laemmli SDS loading buffer (10% glycerol, 5% SDS, 5% β-mercaptoethanol, 0.01% bromophenol blue, and 125 mM TRIS-HCl, pH 6.8) and loaded onto a 12% acrylamide gel (TGX Stain-free Gel FastCast; Bio-Rad Laboratories, CA, USA). After electrophoresis, proteins were transferred to PVDF membranes (Immobilon-P, Millipore, MA, USA) using mini Trans-Blot Electrophoretic transfer cell (Bio-Rad Laboratories, CA, USA). Membranes were then blocked for 1 h at room temperature with Tris-buffered saline containing 5% nonfat dried milk and 0.1% Tween-20 and incubated overnight at 4°C with the following primary polyclonal antibodies: anti-FAAH (CAY-101600, Cayman Chemical, MI, USA) used at 1/1,000, anti-MAGL (MGL-Rb-Af200, Frontier Institute, Hokkaido, Japan) used at 1/1,000, anti-CB_1_ (CB1-Rb-Af380, Frontier Institute, Hokkaido, Japan) used at 1/500, and anti-CB_2_ (ab3561, Abcam, Cambridge, UK) used at 1/200. Membranes were finally incubated with an ECL horseradish peroxidase-linked whole secondary antibody (GE Healthcare UK Limited, Buckinghamshire, UK) used at a 1:5,000 dilution for 2 h at room temperature. Reactive bands were detected by chemiluminescence with the Amersham ECL Prime Western Blotting Detection Reagent (GE Healthcare UK Limited, Buckinghamshire, UK). Images were analyzed with Image Lab software (Bio-Rad Laboratories, CA, USA). Data were calculated as the ratio between the optical densities of the specific protein band and the total protein measured in membranes, and then normalized as percentages over the values of wild-type mice.

### Immunofluorescence

Fixed brains were sliced in coronal sections of 30 μm thick (containing prefrontal cortex, striatum, or hippocampus) in a cryostat (Leica CM3050, Leica, Wetzlar, Germany), collected on antifreeze solution (glycerol/ethylene glycol/PBS; 2:3:5) and stored at −20°C until used. Sections were mounted on gelatin-coated slides and, once adhered, washed with Tris-buffered saline at pH 7.5. Then, sections were permeabilized with Tris-buffered saline containing 0.2% Triton X-100 for 30 min, and unspecificities were blocked with Tris-buffered saline containing 0.1% Triton X-100 and 2% BSA for 1 h. After several washes with Tris-buffered saline, sections were incubated overnight at 4°C with the following primary antibodies: (i) rabbit polyclonal anti-Iba-1 antibody (Wako Chemicals, Richmond, VA, USA) used at 1/500 or (ii) rabbit polyclonal anti-GFAP (Dako Cytomation, Glostrup, Denmark) used at 1/200. Dilutions were carried out in Tris-buffered saline containing 0.1% Triton X-100 and 2% BSA. After incubation, sections were washed in Tris-buffered saline, followed by incubation for 2 h at 37°C with the secondary antibody Alexa Fluor 488 donkey anti-rabbit (Life Technologies, Bleiswijk, The Netherlands) used at 1/200, rendering green fluorescence. Negative control sections were obtained using the same protocol with omission of the primary antibody. A Leica DMRB microscope and a DFC300FX camera (Leica, Wetzlar, Germany) were used for the observation and photography of the slides, respectively. Immunostaining was quantified by measuring the mean density of labeling in the selected area using the ImageJ software (U.S. National Institutes of Health, Bethesda, Maryland, USA, http://imagej.nih.gov/ij/, 1997-2012). For quantification, high-resolution digital microphotographs were taken with the ×20 objective under the same conditions of light, brightness, and contrast. Data were normalized over the values of wild-type mice.

For quantification of cell proliferation in the hippocampal neurogenic niche, immunostaining was carried out following a standard procedure: the sections were incubated with blocking and permeabilization solution containing 0.25% Triton X-100 and 3% BSA in PBS for 3 h at room temperature, and then incubated overnight with the primary antibodies (diluted in the same solution) at 4°C. After thorough washing with PBS, the sections were incubated with fluorochrome-conjugated secondary antibodies diluted in the permeabilization and blocking solution for 3 h at room temperature. After washing with PBS, the sections were mounted on slides with Dako fluorescent mounting medium (Dako, Carpinteria, CA, USA). The following primary antibodies were used: goat anti-GFAP (Ab53554, 1/1,000; Abcam, Cambridge, UK) and rabbit anti-Ki67 (VP-RM04, 1/1,000; Vector Laboratories, Burlingame, CA, USA). 4′,6-Diamidino-2-phenylindole (DAPI, Sigma-Aldrich), at 1/1,000, was also added to the sections during the incubation with the secondary antibodies to counterstain cell nuclei. Quantitative analysis of cell populations was performed by design-based (assumption free, unbiased) stereology using a Leica SP8 (Leica, Wetzlar, Germany) laser scanning microscope and LAS X software. Images were exported as tiffs and adjusted for brightness, contrast, and background, equally for the entire image, using the “levels” tools in Adobe Photoshop without any further modification. All absolute quantifications were performed at ×40 magnification, capturing frames of known volume (typically 100 w × 100 h × 10 d). Density was then normalized for the total volume of the dentate gyrus [subgranule zone (SGZ) + granule cell layer (GCL)] obtained using DAPI staining. For proportional quantifications (with Ki67), representative numbers ranging from 50 to 300 cells of interest were quantified and classified in randomly selected confocal z-stacks from 30 μm below to 30 μm above the GCL using a ×63 oil immersion objective. GCL dispersion was calculated by measuring the thickness of the GCL using DAPI staining for visualization (distance from the hilus to the molecular layer) in three different points in each slice. Cell circularity was measured using the “Circularity” calculation plugin built into ImageJ in the Analyze/Set Measurements and the Measure and Analyze Particles commands after importing the images and selecting at least 50 cells per slice. The command calculates object circularity using the formula: circularity = 4pi(area/perimeter^2^).

### Statistical Analysis

Data were normally distributed (tested with the Shapiro-Wilk normality test) and were assessed, as required (see test used in the legends to figures), by the Student's *t*-test or by one- or two-way (with repeated measures) ANOVA followed by the Bonferroni test, using GraphPad Prism® software (version 5.01; GraphPad Software Inc., San Diego, CA, USA).

## Results

### Behavioral Data

**Figure 1:**
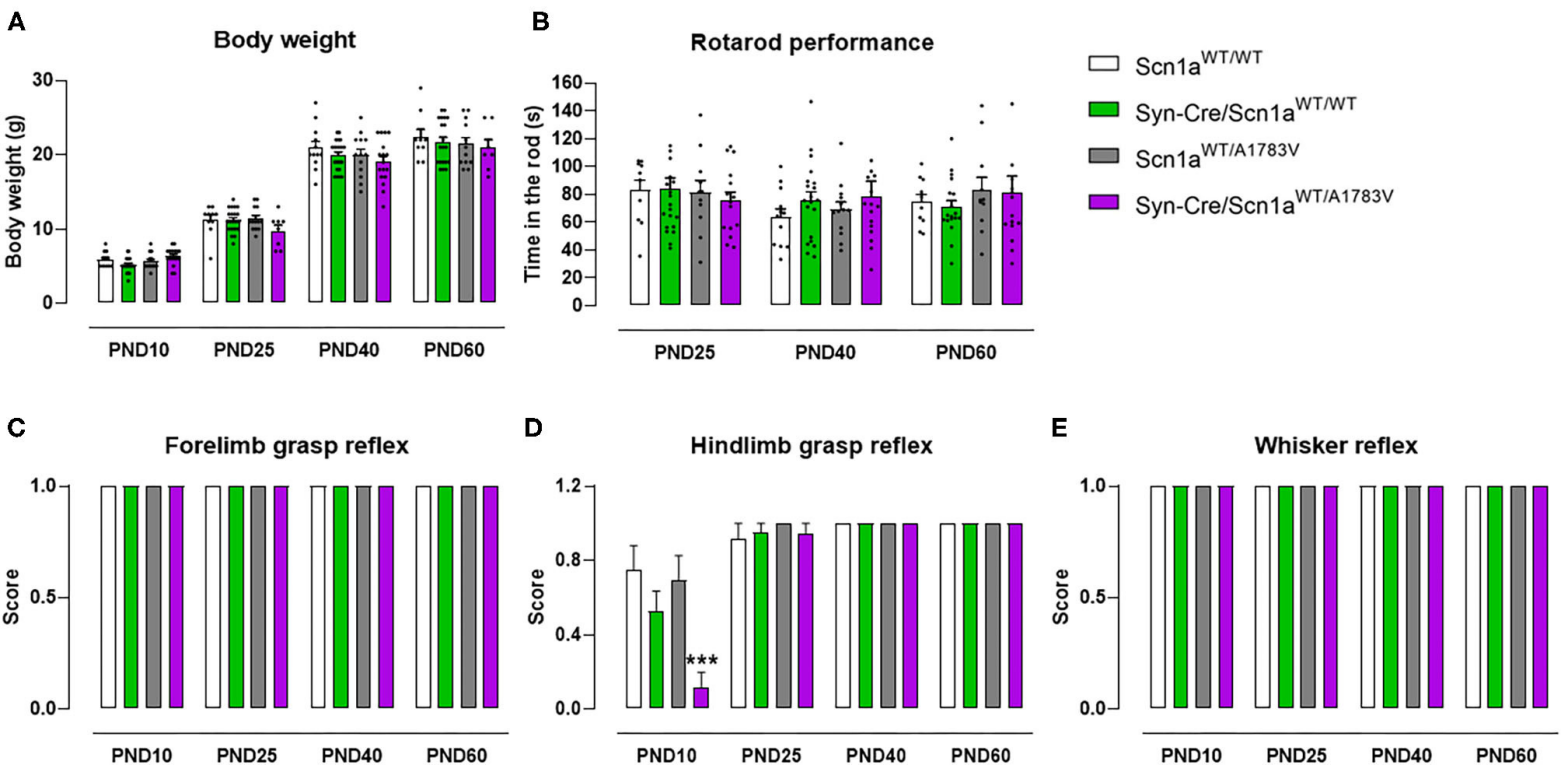
Evolution of body weight, rotarod performance, forelimb and hindlimb grasp reflexes, and whisker reflexes in Syn-Cre/Scn1a^WT/A1783V^ mice and their three control groups (Scn1a^WT/*WT*^, Syn-Cre/Scn1a^WT/WT^, and Scn1a^WT/A1783V^ mice) measured at different postnatal days in the range PND10-PND60. Values are means ± SEM of 12–21 animals per group. Data were assessed by using two-way ANOVA for repeated measures followed by the Bonferroni test (***p < 0.005 vs. the other three groups).

**Figure 2:**
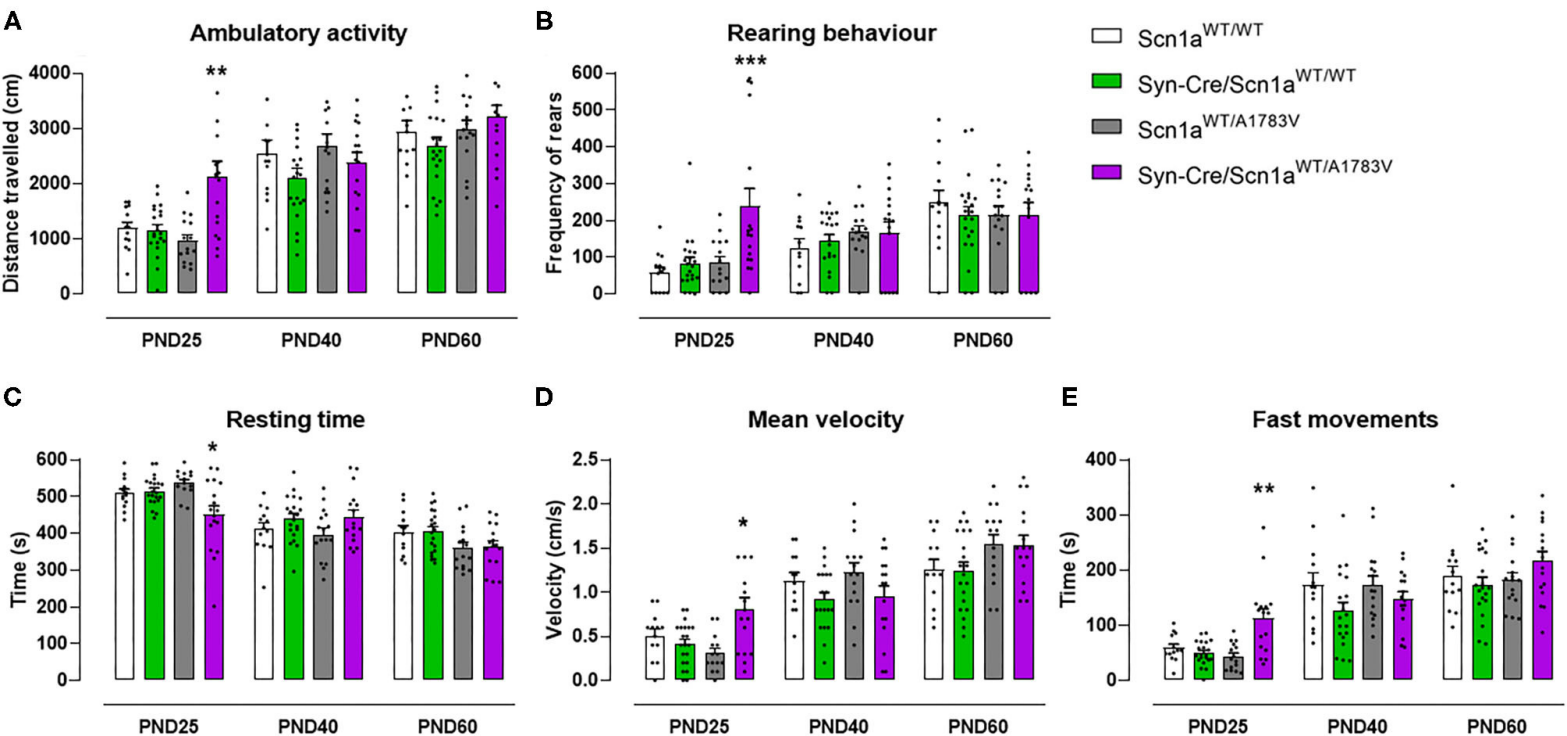
Evolution of different behavioral parameters recorded in the computer-aided actimeter in Syn-Cre/Scn1a^WT/A1783V^ mice and their three control groups (Scn1a^WT/WT^, Syn-Cre/Scn1a^WT/WT^, and Scn1a^WT/A1783V^ mice) measured at different postnatal days in the range PND25–PND60. Values are means ± SEM of 13–21 animals per group. Data were assessed by using two-way ANOVA for repeated measures followed by the Bonferroni test (*p < 0.05, **p < 0.01, ***p < 0.005 vs. the other three groups).

**Figure 3:**
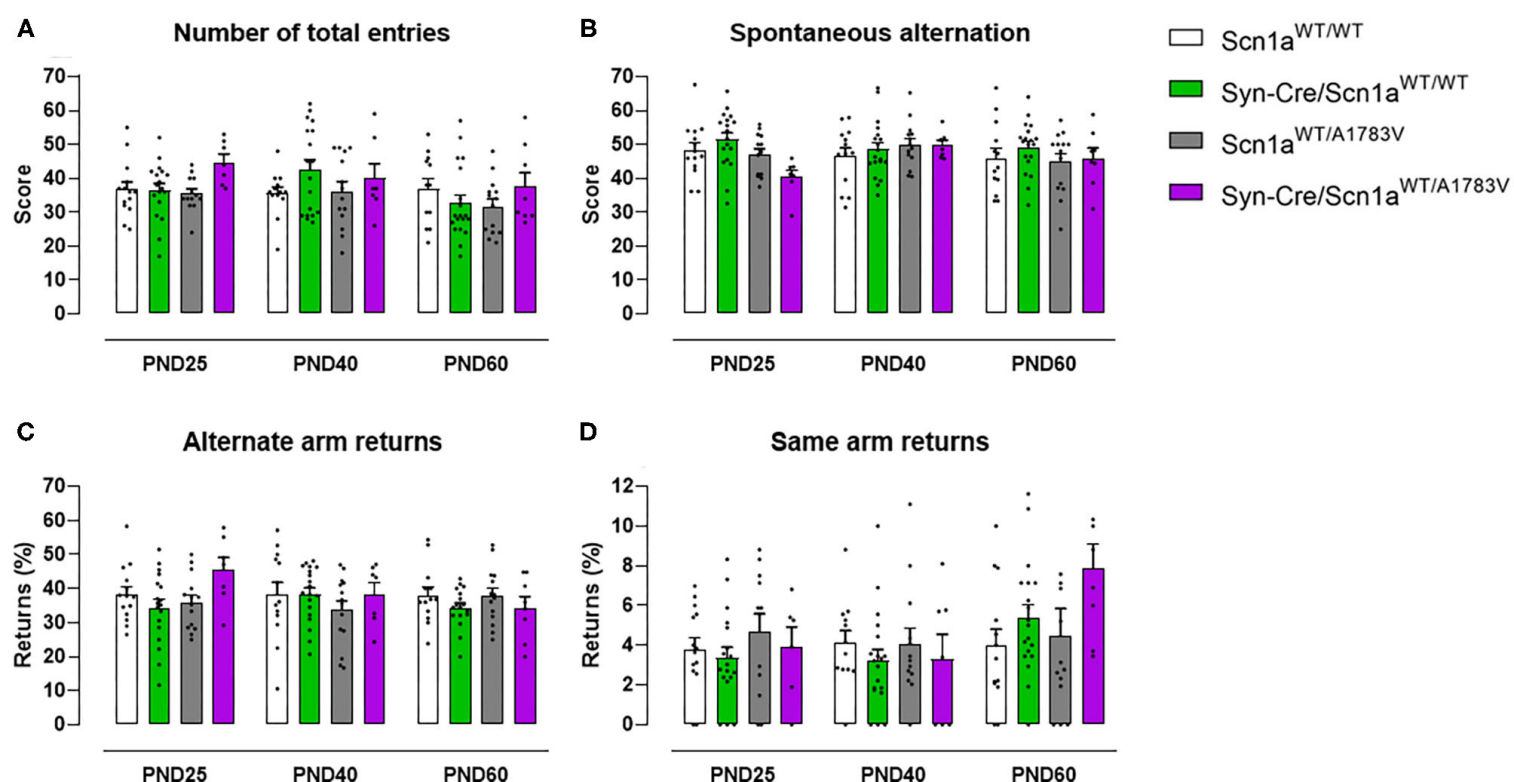
Evolution of different behavioral parameters recorded in the Y-maze in Syn-Cre/Scn1a^WT/A1783V^ mice and their three control groups (Scn1a^WT/WT^, Syn-Cre/Scn1a^WT/WT^, and Scn1a^WT/A1783V^ mice) measured at different postnatal days in the range PND25–PND60. Values are means ± SEM of 13–21 animals per group. Data were assessed by using two-way ANOVA for repeated measures followed by the Bonferroni test.

**Figure 4:**
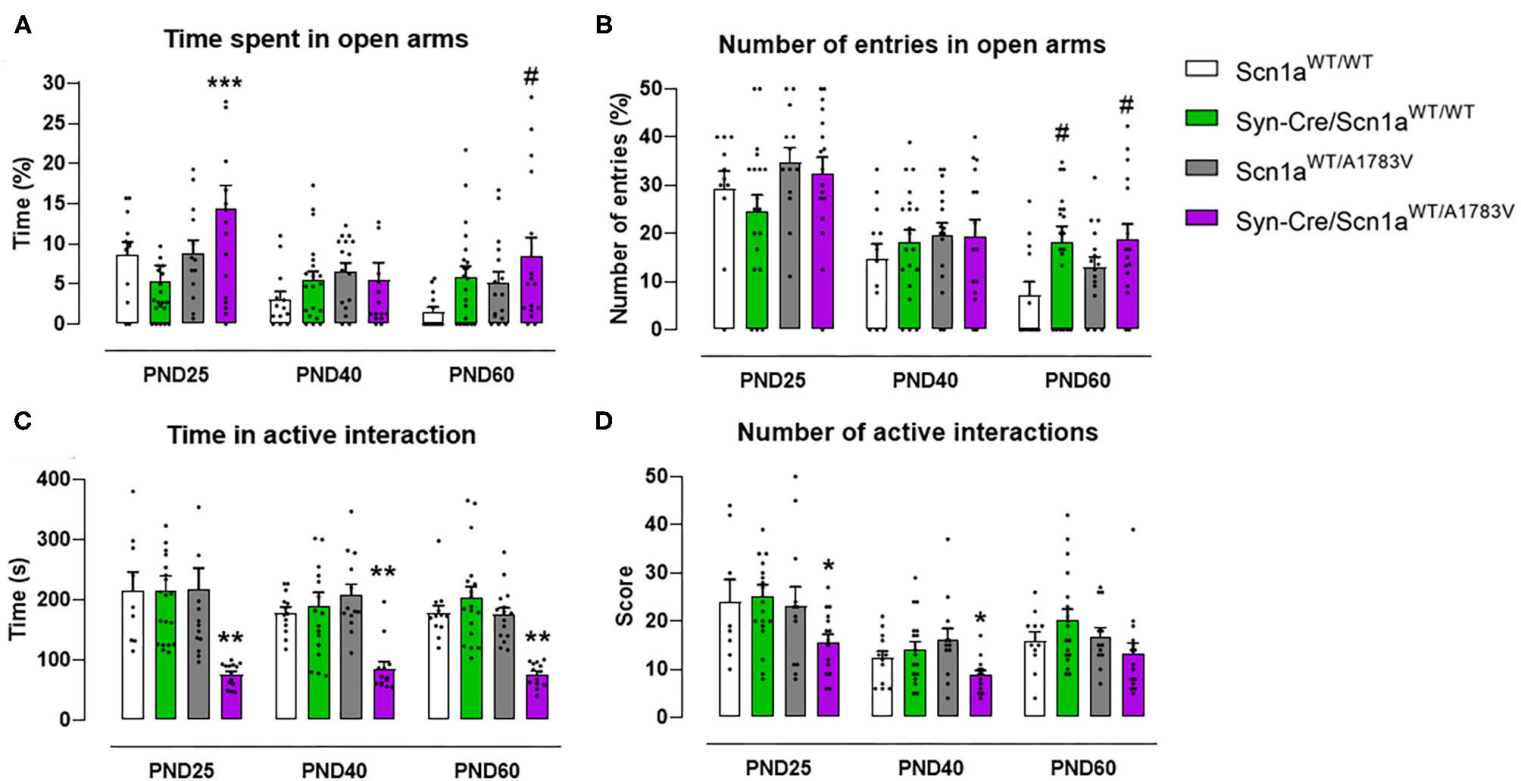
Evolution of different behavioral parameters recorded in the elevated plus maze (upper panels) and in the social interaction test (lower panels) in Syn-Cre/Scn1a^WT/A1783V^ mice and their three control groups (Scn1a^WT/WT^, Syn-Cre/Scn1a^WT/WT^, and Scn1a^WT/A1783V^ mice) measured at different postnatal days in the range PND25–PND60. Values are means ± SEM of 11–21 animals per group. Data were assessed by using two-way ANOVA for repeated measures followed by the Bonferroni test (*p < 0.05, **p < 0.01, ***p < 0.005 vs. control groups; ^#^*p* < 0.05 vs. Scn1a^WT/WT^ group).

**Figure 5:**
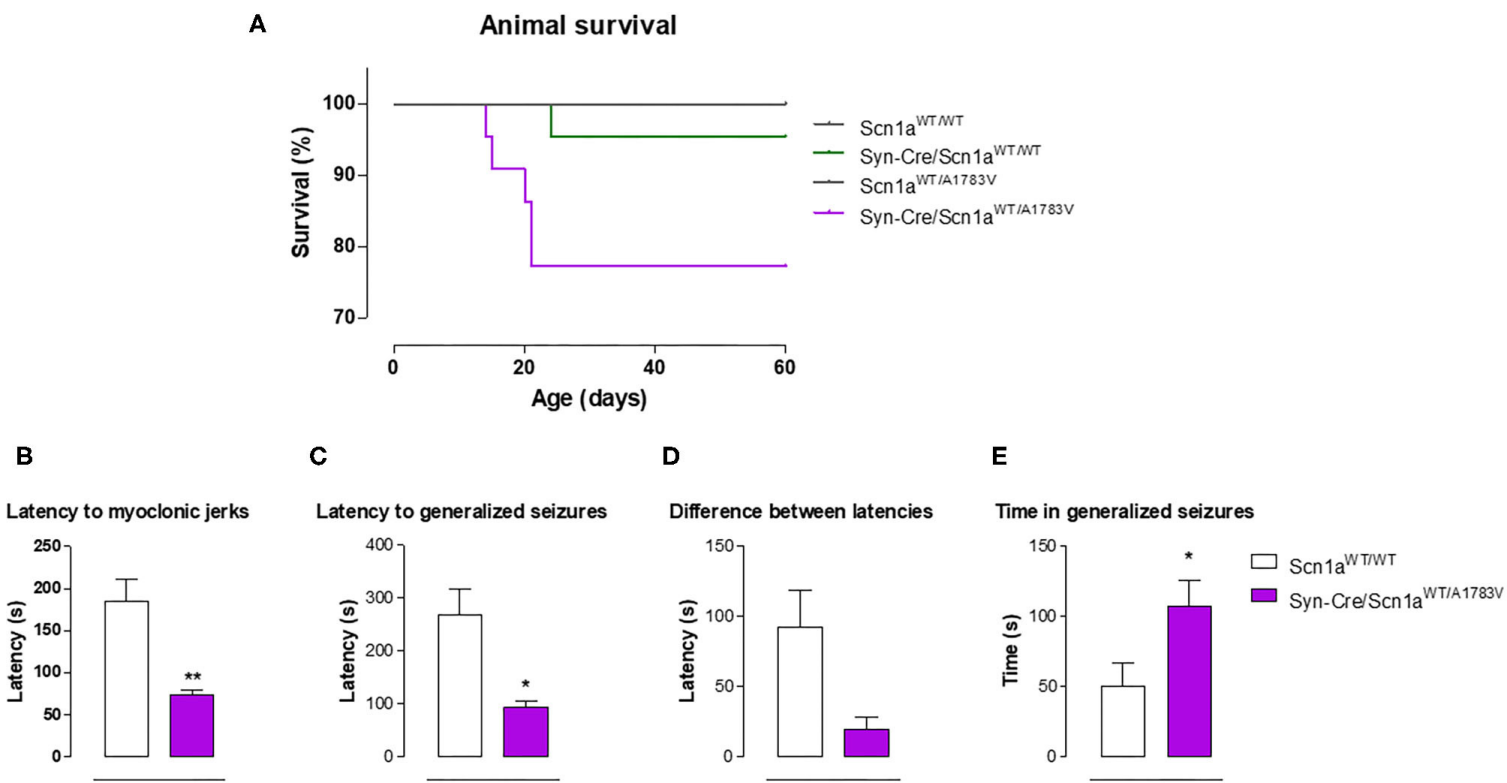
Animal survival in Syn-Cre/Scn1a^WT/A1783V^ mice and their three control groups (Scn1a^WT/WT^, Syn-Cre/Scn1a^WT/WT^, and Scn1a^WT/A1783V^ mice), and seizuring activity in Syn-Cre/Scn1a^WT/A1783V^ mice compared with Scn1a^WT/W^ animals after an acute injection of PTZ at PND25 (lower panels). Values are means ± SEM of six to eight animals per group. Data were assessed by Kaplan-Meier analysis (survival data) and by the Student's *t*-test (PTZ experiment; *p < 0.05, **p < 0.01 vs. the control group).

The first objective (first experiment) of our study was to demonstrate that our mice exhibited behavioral alterations reminiscent of DS. To this end, Syn-Cre/Scn1a^WT/A1783V^ mice and their different controls (Scn1a^WT/WT^, Syn-Cre/Scn1a^WT/WT^, and Scn1a^WT/A1783V^ mice) were subjected to different behavioral tests at some representative postnatal days (PND10–PND60). These data are presented in Figures 1–5. In parallel, their weight was recorded at specific timepoints (Figure 1A) showing differences by age [*F*_(4, 284)_ = 28.07, *p* < 0.0001], but there were no changes by genotype [*F*_(3, 284)_ = 0.23, ns] that may be relevant for the behavioral data.

#### Grasp Reflexes

Syn-Cre/Scn1a^WT/A1783V^ mice exhibited a strong reduction in hindlimb grasp reflex compared with the three control groups [genotype: *F*_(3, 233)_ = 5.25, *p* < 0.005; age: *F*_(3, 233)_ = 46.45, *p* < 0.0001; interaction: *F*_(9, 233)_ = 5.01, *p* < 0.0001; Figure 1D]. Such reduction was not seen in frontlimb grasp (Figure 1C) and whisker reflexes (Figure 1E). This alteration was seen only at PND10, then disappearing at PND25, PND40, and PND60, which should be interpreted as a delay in early motor development of DS newborns.

#### Motor Coordination and Activity

At PND25, mice were analyzed in the rotarod test to measure their motor coordination, but no changes among the four mouse genotypes were detected [genotype: *F*_(3, 172)_ = 0.08, ns; Figure 1B]. Such absence of changes was also evident at further PND40 and PND60 (Figure 1B). Mice were also analyzed at PND25 in a computer-aided actimeter to record some parameters reflecting motor activity. Syn-Cre/Scn1a^WT/A1783V^ mice showed elevated ambulation [genotype: *F*_(3, 185)_ = 5.10, *p* < 0.005; interaction: *F*_(6, 185)_ = 2.79, *p* < 0.05; Figure 2A], frequency of rearing [genotype: *F*_(3, 185)_ = 3.13, *p* < 0.05; interaction: *F*_(6, 185)_ = 2.55, *p* < 0.05; Figure 2B], mean velocity [genotype: *F*_(3, 183)_ = 2.27, *p* = 0.082; interaction: *F*_(6, 183)_ = 3.32, *p* < 0.005; Figure 2D], and time spent in fast movements [genotype: *F*_(3, 185)_ = 5.17, *p* < 0.005; interaction: *F*_(6, 185)_ = 2.73, *p* < 0.05; Figure 2E] compared with the three controls, thus reflecting the occurrence of motor hyperactivity. This was also confirmed by the reduced resting time exhibited by Syn-Cre/Scn1a^WT/A1783V^ mice in the computer-aided actimeter [genotype: *F*_(3, 183)_ = 1.77, *p* = 0.154; interaction: *F*_(6, 183)_ = 3.23, *p* < 0.005; Figure 2C] compared with controls. The computer-aided actimeter was also applied to the same animals when they reached PND40 and PND60, and, despite the progressive elevation in their values of ambulation [age: *F*_(2, 185)_ = 73.6, *p* < 0.0001; Figure 2A], frequency of rearing [age: *F*_(2, 185)_ = 14.5, *p* < 0.0001; Figure 2B], mean velocity [age: *F*_(2, 183)_ = 59.1, *p* < 0.0001; Figure 2D], time spent in fast movements [age: *F*_(2, 185)_ = 60.1, *p* < 0.0001; Figure 2E], and their reduction in the resting time [age: *F*_(2, 183)_ = 59.8, *p* < 0.0001; Figure 2C], no changes were evident in relation with the different genotypes, then indicating that the hyperactivity was, in general, circumscribed to the pre-adolescent period (PND25), as also reported in DS patients (Darra et al., 2019; Verheyen et al., 2019).

#### Spatial Working Memory

Animals were also subjected to the Y-maze, which serves as an index of animal abilities in spatial working memory and spontaneous exploration. Our data at PND25 indicated a subtle worsening in the performance in this test shown by Syn-Cre/Scn1a^WT/A1783V^ mice, apparently visible for the four parameters analyzed (Figures 3A–D). The statistics for the number of total entries in the arms [genotype: *F*_(3, 148)_ = 2.13, *p* = 0.099] and for the % of spontaneous alternation [genotype: *F*_(3, 148)_ = 1.80, *p* = 0.151] confirmed the existence of numerical trends (toward an increase and toward a decrease, respectively) for both parameters in Syn-Cre/Scn1a^WT/A1783V^ mice (Figures 3A,B). However, this did not occur with the statistics for the % of same arm returns [genotype: *F*_(3, 148)_ = 1.66, ns] and for the % of alternate arm returns [genotype: *F*_(3, 148)_ = 0.94, ns], despite apparently similar increases could be appreciated in Syn-Cre/Scn1a^WT/A1783V^ mice (Figures 3C,D). These alterations, as seen with the above behavioral parameters, disappeared when animals became 40 days old (Figures 3A–D), although some of the trends were again visible at PND60, for example, the increase in the % of same arm returns (Figure 3D).

#### Anxiety

Mice were also examined in the elevated plus maze test, which provides information on animal anxiety levels. Our data revealed a lower level in Syn-Cre/Scn1a^WT/A1783V^ mice reflected by a higher % of time spent in open arms [genotype: *F*_(3, 176)_ = 4.17, *p* < 0.01; interaction: *F*_(6, 176)_ = 1.56, *p* = 0.162; Figure 4A] compared with controls, despite that the number of entries in these arms was not statistically significant [genotype: *F*_(3, 177)_ = 2.39, *p* = 0.07; interaction: *F*_(6, 177)_ = 1.43, ns; Figure 4B]. Again, as seen with the above behavioral parameters, these changes disappeared when animals became 40 days old (Figures 4A,B), although some differences were again visible at PND60 for both parameters (Figures 4A,B).

#### Social Interaction

Syn-Cre/Scn1a^WT/A1783V^ mice were also investigated in a social interaction test that may reveal potential autistic-like traits, one of the frequent signs present in DS patients (Skluzacek et al., 2011; Guerrini, 2012). In this test, Syn-Cre/Scn1a^WT/A1783V^ mice spent significantly lower time interacting [genotype: *F*_(3, 185)_ = 26.66, *p* < 0.0001; Figure 4C], as well as reducing their number of contacts [genotype: *F*_(3, 185)_ = 5.042, *p* < 0.005; Figure 4D], with a novel unfamiliar partner compared with the other groups, supporting a deficit in social interaction. Such deficit was found at PND25 and, contrary to the other behavioral impairments found at this age in DS mice, it was also found at PND40 and PND60, as reflecting by the lack of statistical significance for the age × genotype interaction for both parameters [time in active interaction: *F*_(6, 185)_ = 0.504, ns; number of active interactions: *F*_(6, 185)_ = 0.472, ns; Figures 4C,D].

#### Seizuring Activity

Syn-Cre/Scn1a^WT/A1783V^ mice frequently show spontaneous convulsions (see video in the Supplementary File 2), although these are difficult to record and quantify. An option to record the seizuring activity of Syn-Cre/Scn1a^WT/A1783V^ mice is to determine their vulnerability to proconvulsant agents, so, in the second experiment, we compared seizuring activity of Syn-Cre/Scn1a^WT/A1783V^ mice with their controls (only wild-type mice with no Cre expression: Scn1a^WT/WT^) once they were acutely injected with the proconvulsant agent PTZ at PND25. Our data demonstrated that DS mice responded faster and more intensely to PTZ compared with controls, as reflected in their reduction in the latency to myoclonic jerks and generalized seizures, and the differences between both latencies (trend as *p* = 0.058), as well as in the higher time spent in seizures (Figures 5B–E).

### Survival Data

In the first experiment, in which Syn-Cre/Scn1a^WT/A1783V^ mice and their three controls were recorded between PND10 and PND60 for detecting possible behavioral impairment (Figures 1–5), mice were also used for measuring animal survival. Our data indicated that survival in Syn-Cre/Scn1a^WT/A1783V^ mice was significantly shorter compared with the other groups, with almost one of four animals (25%) dying before PND60 and no death (or only a case) in the other three genotypes (Figure 5A). It is also important to remark that, despite the reduction in survival, this exceeded 60 days of life and was significantly much more moderate compared with the murine model (also based on the A1783V mutation but expressed in all body cells) used by Ricobaraza et al. (2019), whose mortality reached up to 75% at similar ages. This makes our model useful for long-term studies in this disease, in particular for exploring the comorbidities associated with DS, which cannot be investigated in models based on complete deletion of *Scn1a* gene due to premature mortality [they die before 2 weeks of age (Ogiwara et al., 2007)], and can only be partially studied in models based on heterozygous deletion, which, although present delayed mortality, reach elevated rates (they die at 3–12 weeks of age, having only a 20% of long-term survival) at the ages more adequate for investigating long-term comorbidities (Yu et al., 2006).

### Biochemical and Histopathological Data

**Figure 6:**
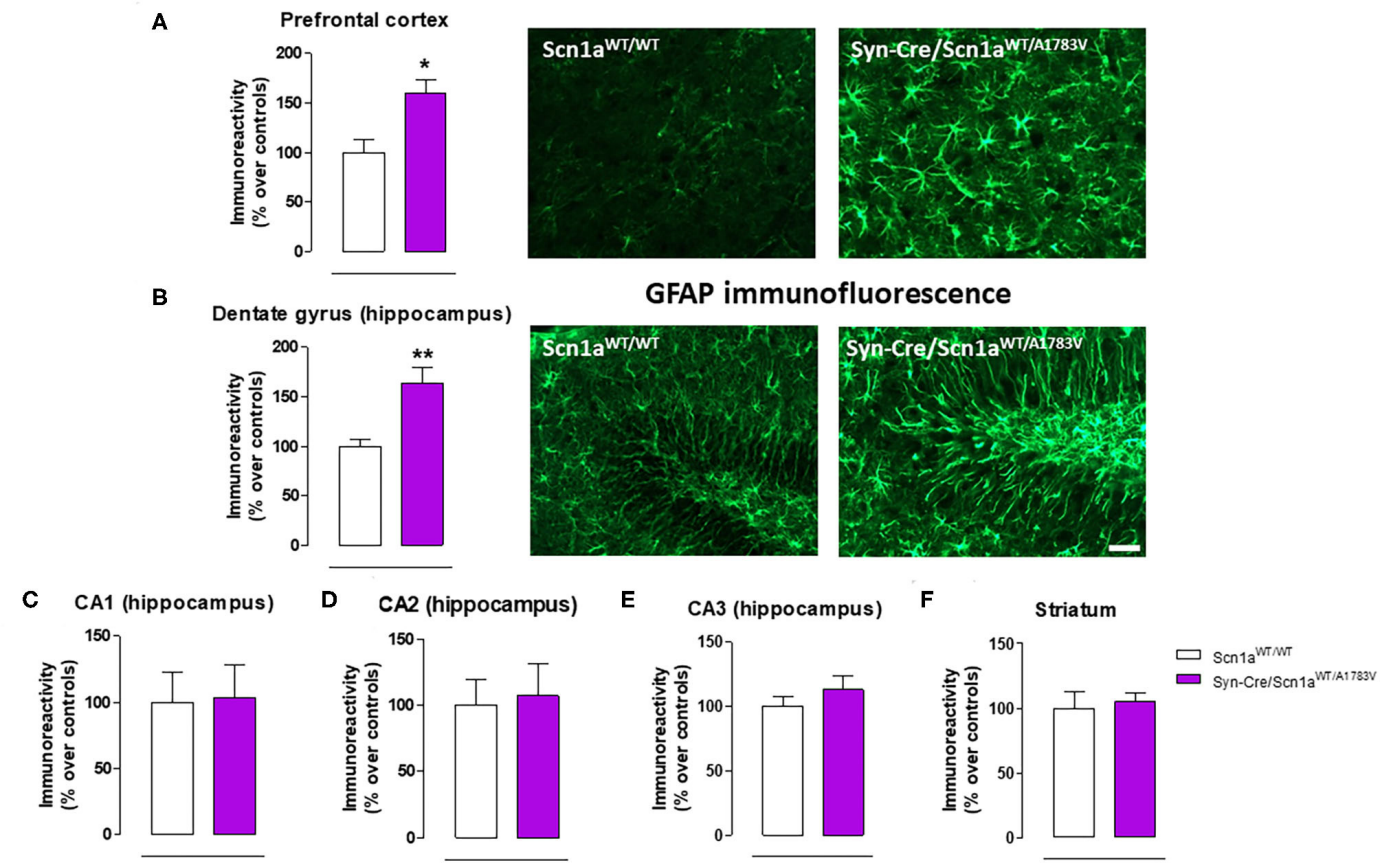
Immunoreactivity for the astroglial marker GFAP measured by immunofluorescence in different CNS structures, including representative immunofluorescence images in the prefrontal cortex and the hippocampal dentate gyrus of Syn-Cre/Scn1a^WT/A1783V^ mice compared with Scn1a^WT/W^ animals at PND25. Values are % over controls and correspond to means ± SEM of five to six animals per group. Data were assessed by the Student's *t*-test (*p < 0.05, **p < 0.01 vs. the control group). Scale bar = 100 μm.

**Figure 7:**
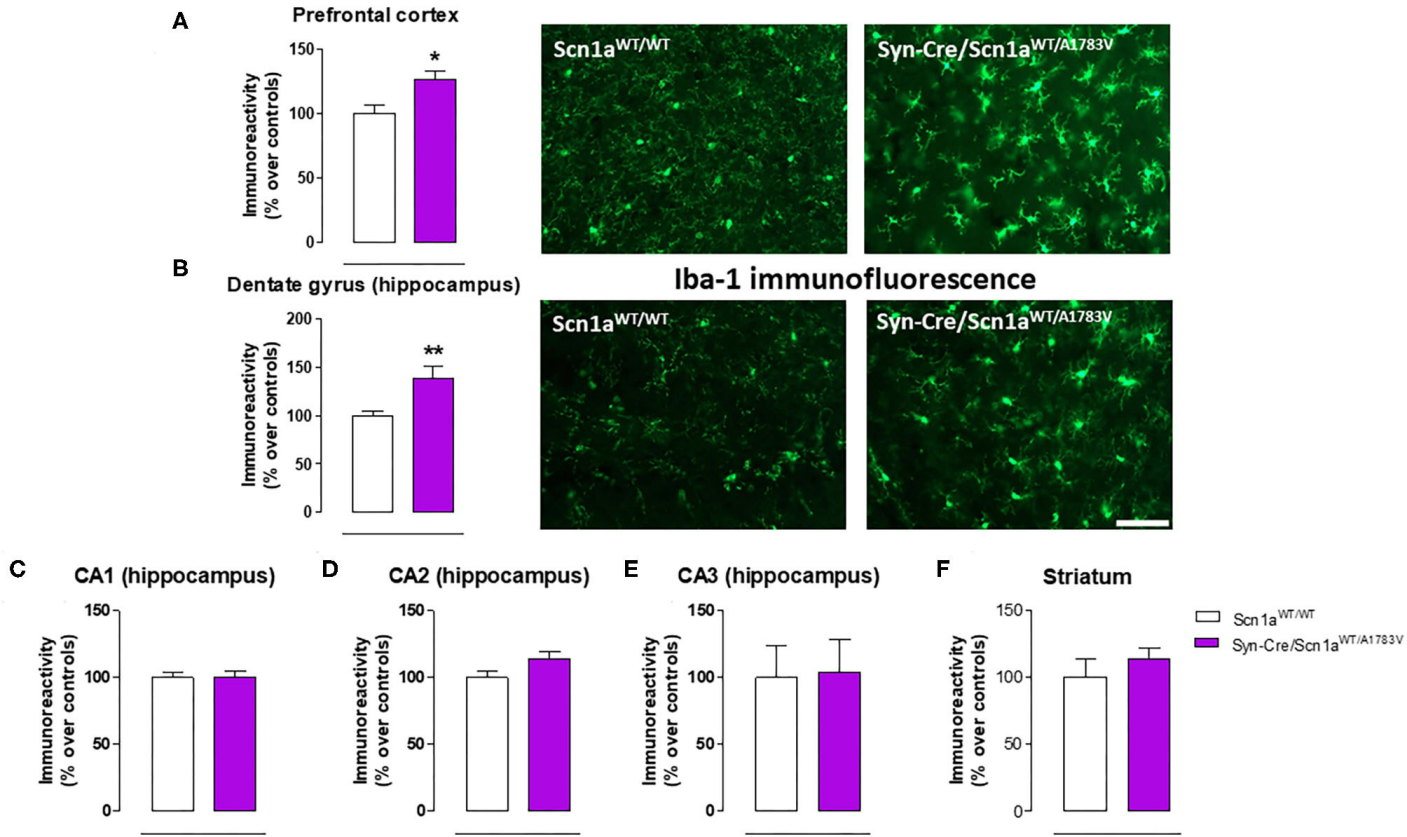
Immunoreactivity for the microglial marker Iba-1 measured by immunofluorescence in different CNS structures, including representative immunofluorescence images in the prefrontal cortex and the hippocampal dentate gyrus, of Syn-Cre/Scn1a^WT/A1783V^ mice compared with Scn1a^WT/W^ animals at PND25. Values are % over controls and correspond to means ± SEM of five to six animals per group. Data were assessed by the Student's *t*-test (*p < 0.05, **p < 0.01 vs. the control group). Scale bar = 100 μm.

**Figure 8:**
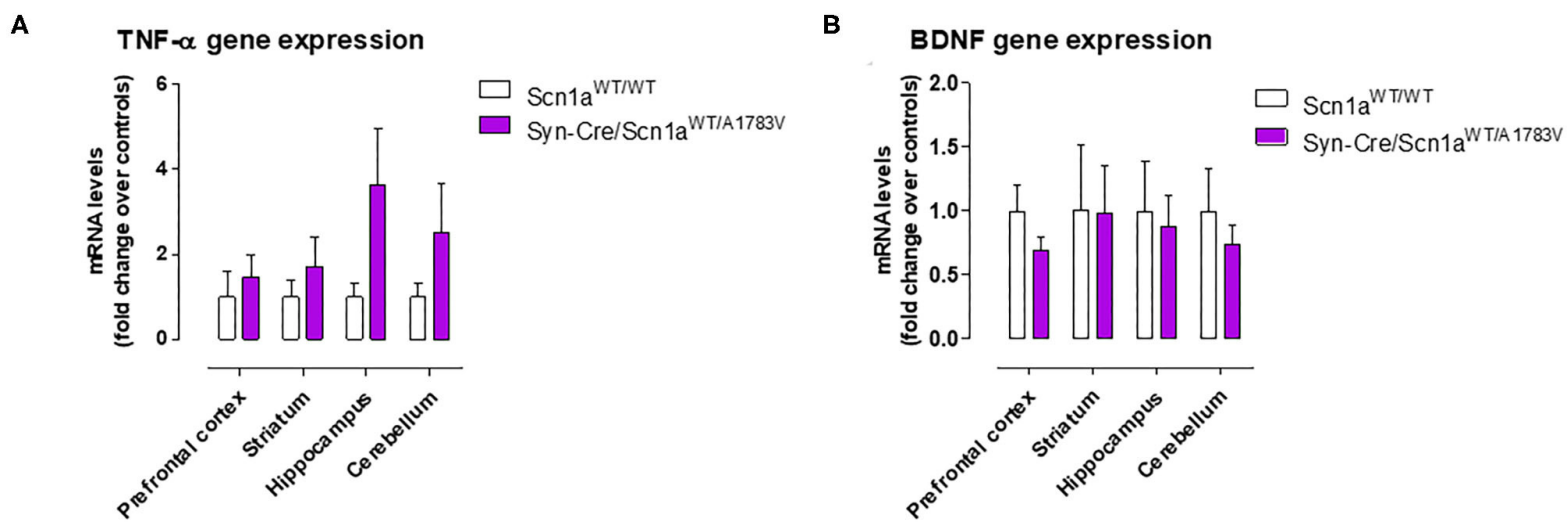
Gene expression for TNF-α and BDNF measured by qPCR in different CNS structures of Syn-Cre/Scn1a^WT/A1783V^ mice compared with Scn1a^WT/W^ animals at PND25. Values are fold of change over controls and correspond to means ± SEM of five to six animals per group. Data were assessed by the Student's *t*-test.

**Figure 9:**
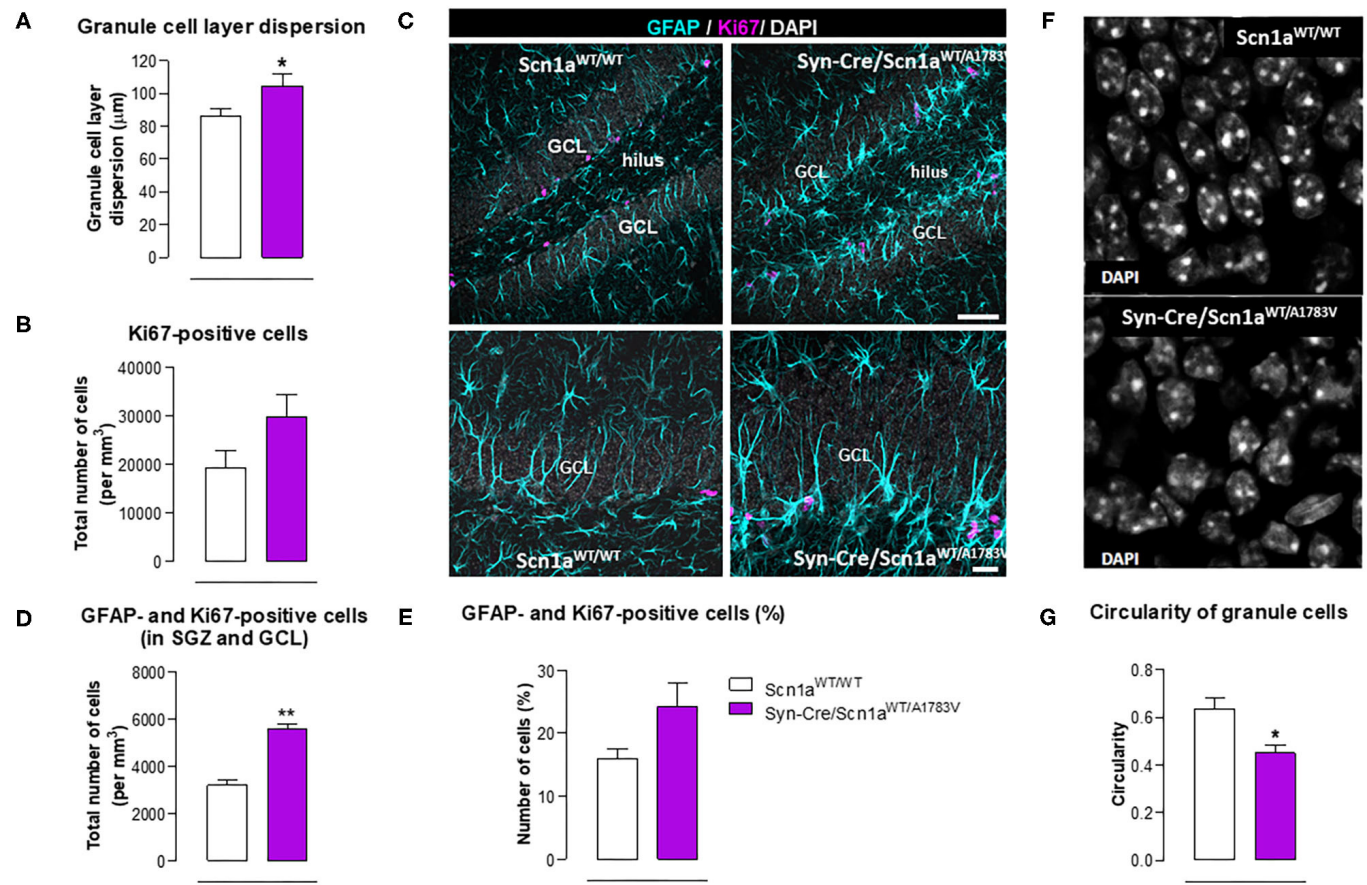
Double immunofluorescence analysis of Ki67 and GFAP in the hippocampal dentate gyrus, including representative immunofluorescence images, of Syn-Cre/Scn1a^WT/A1783V^ mice compared with Scn1a^WT/W^ animals at PND25. Values are means ± SEM of six animals per group. Data were assessed by the Student's *t*-test (*p < 0.05, **p < 0.01 vs. the control group). Scale bar for double labeling = 50 μm (top panels) and 20 μm (bottom panels).

**Figure 10:**
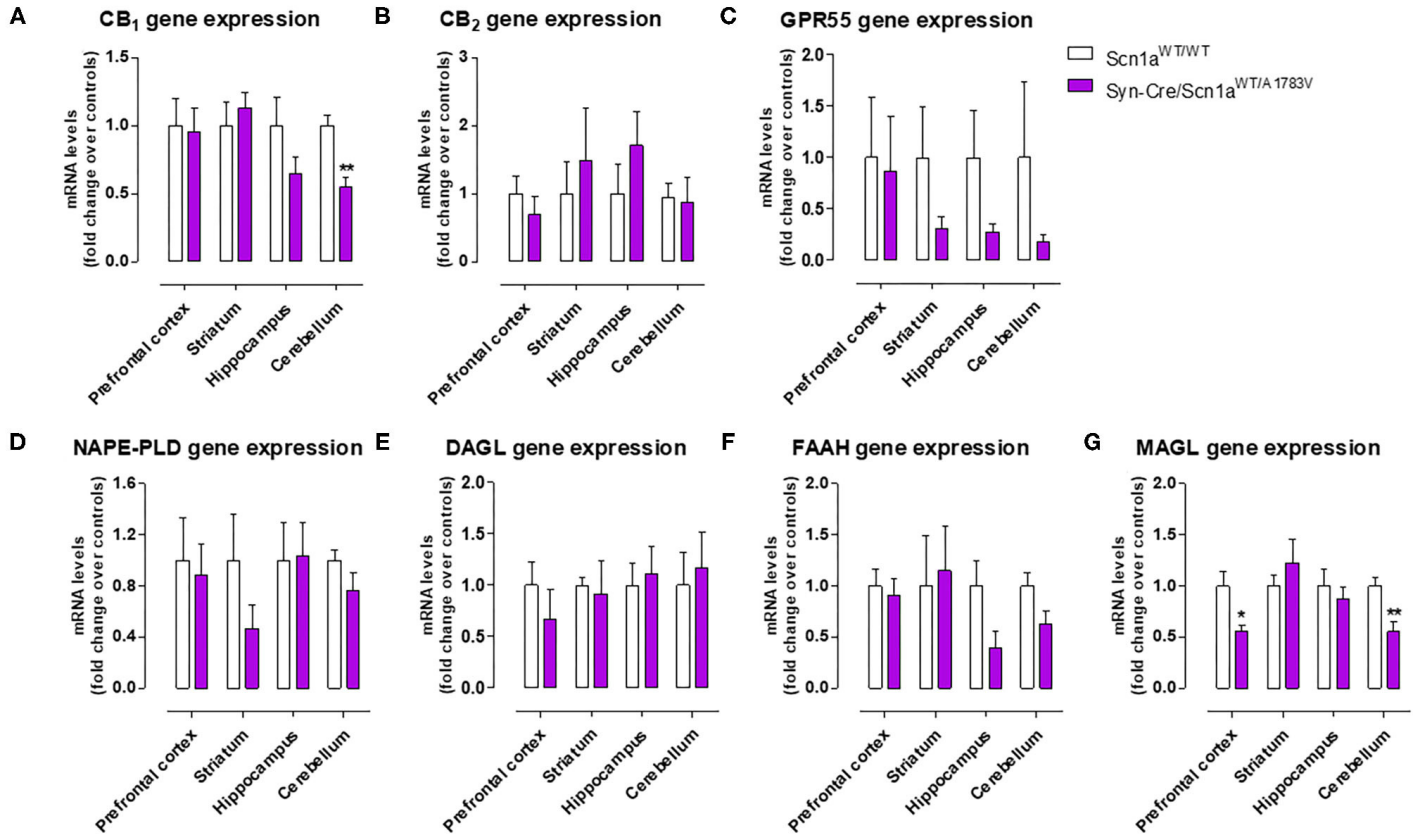
Gene expression for the CB_1_, CB_2_, and GPR55 receptors and the NAPE-PLD, DAGL, FAAH, and MAGL enzymes measured by qPCR in different CNS structures of Syn-Cre/Scn1a^WT/A1783V^ mice compared with Scn1a^WT/W^ animals at PND25. Values are fold of change over controls and correspond to means ± SEM of five to six animals per group. Data were assessed by the Student's *t*-test (*p < 0.05, **p < 0.01 vs. the control group).

**Figure 11:**
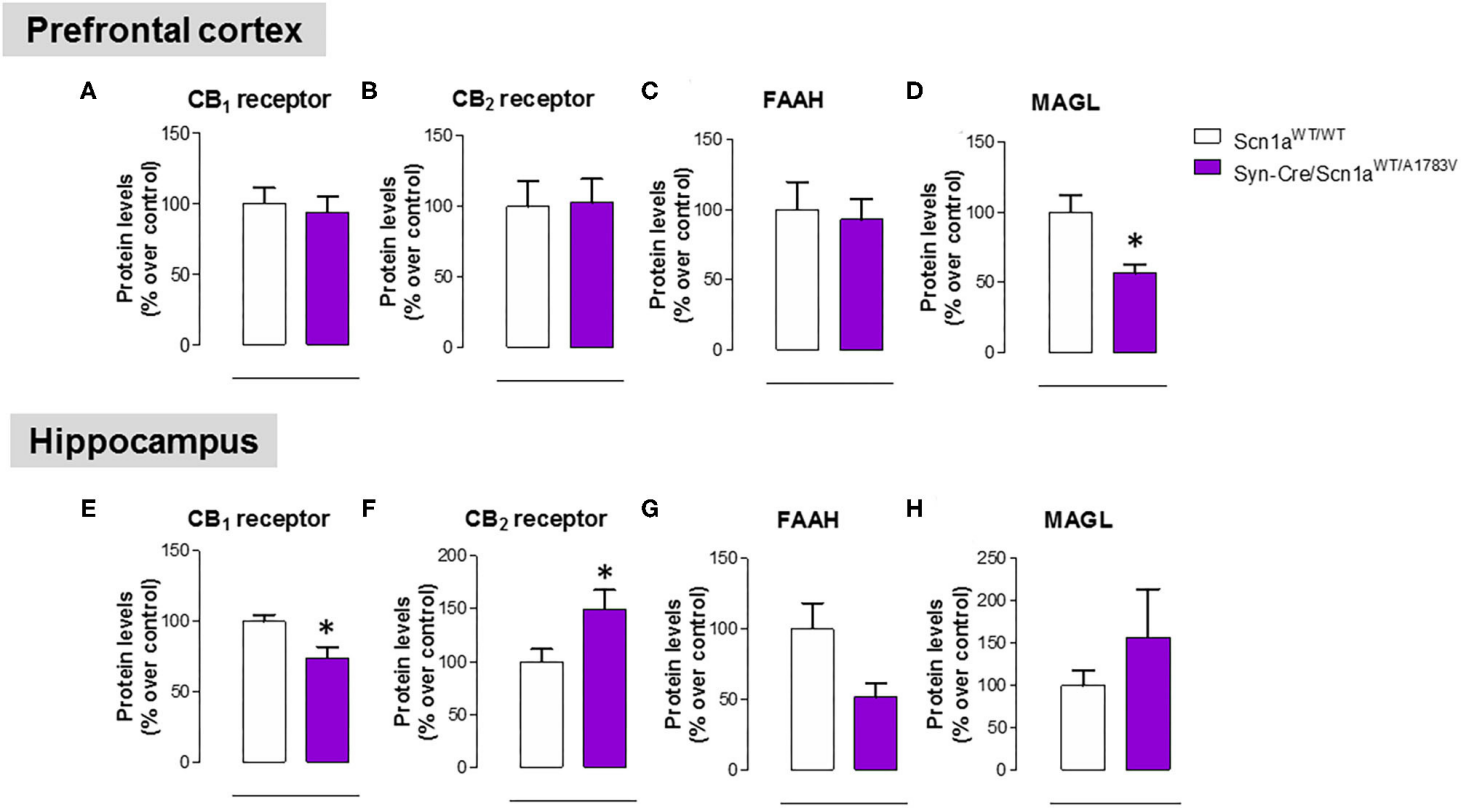
Protein levels for the CB_1_ and CB_2_ receptors and the FAAH and MAGL enzymes measured by western blot in the prefrontal cortex and the hippocampus of Syn-Cre/Scn1a^WT/A1783V^ mice compared with Scn1a^WT/W^ animals at PND25. Values are % over controls and correspond to means ± SEM of five to six animals per group. Data were assessed by the Student's *t*-test (*p < 0.05 vs. the control group).

In the case of animals of the second experiment (excluding those used for PTZ injections), they were used to collect their neural tissues at the PND25, which were used to analyze some inflammation-related markers in specific CNS areas whose alteration could be related to behavioral changes seen during the progression of the pathological phenotype in Syn-Cre/Scn1a^WT/A1783V^ mice. These tissues were also used for analyzing the hippocampal neurogenesis, as well as to determine the status of different endocannabinoid elements. These data are presented in Figures 6–11.

#### Glial Reactivity

Given that the occurrence of glial reactivity and local neuroinflammatory events in infant epileptic conditions is well documented (Boer et al., 2010; Iyer et al., 2010; Koh, 2018), we wanted to investigate whether this happens in our DS mice at the age at which the behavioral impairment was maximal (PND25). Our data demonstrated elevated glial reactivity, reflected in both higher GFAP (Figure 6) and Iba-1 (Figure 7) immunoreactivity and also in morphological changes (shift to an activated state) in GFAP- or Iba-1-positive cells, in the prefrontal cortex (Figures 6A, 7A) and the hippocampal dentate gyrus (Figures 6B, 7B) of Syn-Cre/Scn1a^WT/A1783V^ mice compared with controls. By contrast, such astrogliosis and microgliosis were not found in the different subfields (CA1, CA2, and CA3) of the hippocampal Ammon's horn or in the striatum (Figures 6C–F, 7C–F). In parallel, we also measured gene expression of the proinflammatory cytokine TNF-α, which was apparently elevated in different CNS structures of Syn-Cre/Scn1a^WT/A1783V^ mice, in particular in the hippocampus (*p* = 0.065), although these differences did not reach statistical significance in any case, likely due to a high variation in these mice (Figure 8A). It is important to remark that for these data, only tissues from Syn-Cre/Scn1a^WT/A1783V^ mice and the control animals having expression of a wild-type *SCN1A* gene and no Cre recombinase expression (Scn1a^WT/WT^ mice) were analyzed. We excluded the analyses of the other two control genotypes (Syn-Cre/Scn1a^WT/WT^ and Scn1a^WT/A1783V^ mice) given that no differences among the three control genotypes were found in the behavioral recording. To confirm this fact also in the neuropathological data, we carried out a few analyses using GFAP and Iba-1 immunofluorescence in the three control genotypes, whose results (similar in the three genotypes) are shown in the Supplementary File 3.

#### Neurogenesis Dysregulation

Epilepsy has also been associated with alterations in hippocampal neurogenesis in the dentate gyrus (Bielefeld et al., 2019; Chen et al., 2020), and this was also investigated in our Syn-Cre/Scn1a^WT/A1783V^ mice. We found a higher granule cell dispersion (Figure 9A) and a trend toward an increase (*p* = 0.13) in the number of positive cells for Ki67, a marker of proliferating cells (Figure 9B), supporting the idea of an elevated mobilization of progenitor cells in Syn-Cre/Scn1a^WT/A1783V^ mice. Using double-labeling analyses with Ki67 and GFAP in the GCL and the SGZ (Figure 9C), we found that the number of positive cells for both markers was higher in Syn-Cre/Scn1a^WT/A1783V^ mice [both as total (*p* < 0.01) and as % (trend: *p* = 0.106)] compared with Scn1a^WT/WT^ mice (Figures 9D,E). Cell proliferation was mostly restricted to the SGZ, and in other models of epilepsy, including mesial temporal lobe epilepsy (MTLE) (Sierra et al., 2015) and other inducible model of DS (Martín-Suárez et al., 2020), neural stem cells (NSCs) rather than astrocytes have been found to proliferate in higher number (Sierra et al., 2015; Martín-Suárez et al., 2020). This alteration correlates with a phenotypical change consisting in the transformation into a reactive-like phenotype of NSCs which can also be observed in our model (Figure 9C). Because of the agreements with previous reports, we interpret these data as indicating a reactive neurogenic response likely participating to reactive gliogenesis (Sierra et al., 2015; Martín-Suárez et al., 2020). We also found that neurons forming the GCL had altered nuclear morphology in the DS mice. Instead of the typical round nuclei of the normal mice, in the DS, nuclei presented an irregular contour with indentations. The measurement of nuclei circularity showed a significant decrease in the DS mice (Figures 9F,G). The cause of this morphological transformation remains unknown, but it has been related to cell damage as it is found in different pathologies.

BDNF gene expression was also measured in the hippocampus and also in other CNS structures (prefrontal cortex, striatum, and cerebellum), but no differences between Syn-Cre/Scn1a^WT/A1783V^ mice and controls were detected, with the only exception of a trend toward a decrease seen in the prefrontal cortex (Figure 8B).

#### Endocannabinoid Dysregulation

As mentioned above, neural tissues collected in the second experiment were also used for determining the status of different elements of the endocannabinoid signaling, which may serve as potential targets for a cannabinoid-based therapy in DS (Rubio et al., 2016; Gray and Whalley, 2020). The objective of these analyses was to detect a possible dysregulation in this signaling system in different CNS structures of Syn-Cre/Scn1a^WT/A1783V^ mice, following previous data described in lymphocytes of DS patients (Rubio et al., 2016), which may explain the expected efficacy of cannabinoids as disease modifiers in DS. Our data confirmed this possible dysregulation although this would be restricted to specific CNS structures. Thus, our qPCR analysis demonstrated that the CB_1_ receptor was downregulated in the cerebellum, with a small trend toward a decrease in the hippocampus (*p* = 0.179) of Syn-Cre/Scn1a^WT/A1783V^ mice (Figure 10A), which was corroborated by western blot analysis (Figure 11E). No changes were found in the prefrontal cortex and the striatum (Figures 10A, 11A). With regard to the CB_2_ receptor, we did not find any changes with qPCR in the different structures analyzed in Syn-Cre/Scn1a^WT/A1783V^ mice (Figure 10B), whereas we found elevated CB_2_ receptor levels measured by western blot in the hippocampus (Figure 11F) but not in the prefrontal cortex (Figure 11B). We also analyzed the orphan GPR55 receptor, which was proposed as the target for the benefits of CBD against seizures and autistic signs in the study by Kaplan et al. (2017) conducted in an experimental model of DS. Using qPCR analysis, we detected some trends toward a decrease in the striatum (*p* = 0.2), hippocampus (*p* = 0.15), and cerebellum (*p* = 0.34) of Syn-Cre/Scn1a^WT/A1783V^ mice, which did not reach statistical significance due to high variability, in particular in Scn1a^WT/WT^ mice (Figure 10C). With regard to the endocannabinoid-inactivating enzymes, we found a reduction in MAGL-mRNA in the cerebellum and prefrontal cortex of Syn-Cre/Scn1a^WT/A1783V^ mice, which was confirmed at the protein level only in the case of the prefrontal cortex (Figure 11D). We did not found any changes in the striatum, measured by qPCR (Figure 10G), and in the hippocampus, measured by qPCR (Figure 10G) and western blot (Figure 11H). The other major endocannabinoid-inactivating enzyme, FAAH, was not modified in the prefrontal cortex (Figures 10F, 11C) and the striatum (Figure 10F) of Syn-Cre/Scn1a^WT/A1783V^ mice, although a trend toward a decrease in the qPCR analysis could be appreciated in the cerebellum (*p* = 0.068) and the hippocampus (*p* = 0.062) (Figure 10F), in this last case also found with the western blot analysis (*p* = 0.057) (Figure 11G). Lastly, the expression of the enzymes that synthesize endocannabinoids, NAPE-PLD and DAGL, was not found to be altered in Syn-Cre/Scn1a^WT/A1783V^ mice (Figures 10D,E). Representative blots of all proteins analyzed are shown in the Supplementary File 4.

## Discussion

As indicated and justified in the “**Introduction**,” our study has been conducted with heterozygous conditional knock-in mice carrying a missense mutation (A1783V) in *Scn1a* gene, which were crossed with mice expressing Cre-recombinase linked to synapsin-1 promoter, then generating Syn-Cre/Scn1a^WT/A1783V^ mice bearing the A1783V mutation in Nav1.1 protein expressed exclusively in CNS neurons. Our first objective was to demonstrate that they recapitulate most of the cardinal neurological signs seen in DS patients, including the long-term comorbidities. Our data confirmed that these animals showed cerebral hyperexcitability manifested by spontaneous seizures and, more specifically, by a greater sensitivity to the proconvulsant agent PTZ, showing, in particular, a lower latency to seizuring, as also found in other DS models (Ogiwara et al., 2007; Tai et al., 2014). Our animals also presented some important DS comorbidities, also found in other DS models (Cheah et al., 2012; Han et al., 2012; Tatsukawa et al., 2018; Stein et al., 2019), as hyperactivity (with no changes in motor coordination), a subtle memory impairment, apparently less anxiety, and, in particular, an altered social behavior reflecting possibly the occurrence of autistic traits. All these behavioral signs, which represent some important features present in patients (Skluzacek et al., 2011; Guerrini, 2012; Darra et al., 2019), were found in Syn-Cre/Scn1a^WT/A1783V^ mice at PND25, although we assume that they may be present at earlier ages at which the performance of these tests is not possible due to animal immaturity. This assumption is based on the detection of changes in hindlimb grasp reflexes at PND10, which likely indicates that these mice apparently have a delayed motor development as shown to occur in patients (Verheyen et al., 2019). We also investigated the mortality in our mice. As mentioned above, models based on the homozygous (Ogiwara et al., 2007) and heterozygous [only 20% of long-term survival (Yu et al., 2006)] deletion of *Scn1a* gene have been associated with high mortality, in particular when the gene deletion was complete and carried out in mice with the C57BL/6 background, which results in extremely premature death [around 2 weeks after birth (Ogiwara et al., 2007)]. Models based on the widespread A1783V mutation of the Nav1.1 channel also reduce the survival of animals from PND20 reaching up to 75% at PND40 (Ricobaraza et al., 2019), and this also happens when the mutation is expressed exclusively in GABAergic neurons [100% of mice died prior to PND25 (Kuo et al., 2019)], making it difficult to extend the period under investigation to juvenile and adult ages. Interestingly, when the mutation of the channel is not exclusive of GABAergic neurons, but expressed in different CNS neurons, as is the case in our model, the survival reaches up to 75% of the animals after the PND20 remaining unaltered up to PND60. This higher survival of our animals facilitates to confirm the persistence in these animals of some of the comorbidities found at PND25, in our case, in particular the autistic traits seen in the social interaction test, which still persist at PND40 and PND60, and also some signs detected in the elevated plus maze test, which disappeared at PND40 but were again visible at PND60. By contrast, signs as hyperactivity were only visible at PND25 disappearing in the following ages investigated.

Our second objective in this study was to explore the neuropathological alterations associated in CNS structures with these behavioral anomalies. We concentrated the study in PND25, which was the most affected age in Syn-Cre/Scn1a^WT/A1783V^ mice and paid emphasis on local inflammatory events in view of the contribution that these events appear to have in the DS pathogenesis as indicated in recent studies (Boer et al., 2010; Iyer et al., 2010; Koh, 2018). Our data confirmed two CNS structures significantly altered, the prefrontal cortex and the hippocampal dentate gyrus, which may explain the emotional and memory impairments detected in the behavioral tests, but not in other areas such as the striatum despite the changes found in motor activity. In these two areas, we detected elevated reactive astrogliosis (labeled with GFAP) and microgliosis (labeled with Iba-1), associated, in the case of the hippocampus, with elevated levels of TNF-α mRNA levels, which support the occurrence of local inflammatory events that may be, in part, responsible of the long-term deficits observed in the disease. Hippocampal postnatal and adult neurogenesis is greatly modified by seizures (Bielefeld et al., 2019; Chen et al., 2020). We investigated in particular the response of hippocampal NSCs in the dentate gyrus, as reports from the last years show how this normally neurogenic cell type transforms into a reactive phenotype (reactive NSCs) with abnormal morphology and increased mitotic activity that contributes to reactive gliogenesis (Sierra et al., 2015; Muro-García et al., 2019). These alterations have also been reported very recently to take place in other murine models of DS (Martín-Suárez et al., 2020) and could be potentially responsible, at least in part, of cognitive deficits associated with these conditions. In our study, we detected a trend toward elevated cell proliferation in Syn-Cre/Scn1a^WT/A1783V^ mice, which corresponded more to cell division of GFAP-positive cells rather than to neurons. We hypothesize that these are NSCs rather than astrocytes as this cell type was not found to enter cell division in the studies mentioned above and cell proliferation was restricted to the SGZ and GCL in our work. As normally NSCs account for a small proportion of dividing cells in the dentate gyrus, a significant increase in NSC activation is compatible with a comparatively smaller increment in overall cell division. The data presented herein suggest that, in our model, NSCs could also contribute to reactive astrogliosis found in the hippocampal dentate gyrus which might be at the expense of neurogenesis in Syn-Cre/Scn1a^WT/A1783V^ mice as a direct response to seizures. Alternatively, the response of the hippocampal dentate gyrus in our mice could be secondary and attributed to the extreme inflammatory events and glial reactivity generated by the highly seizuring activity occurring in these mice. Further studies centered in hippocampal neurogenesis will help to clarify these points. Related to gliosis in the dentate gyrus, we found also granule cell dispersion, normally found in focal epilepsies such as MTLE (Sierra et al., 2015; Muro-García et al., 2019). Finally, we found abnormal morphology of the nuclei of neurons in the GCL whose cause is still unknown but have been proposed to be associated with altered lamin expression (Goldman et al., 2004; Vergnes et al., 2004).

As mentioned in the “**Introduction**,” cannabinoids, in particular CBD, have recently been proposed as potential antiseizuring agents in DS and other similar infantile syndromes based on successful clinical trials conducted with Epidiolex® (pure CBD) in children affected by these syndromes (Devinsky et al., 2016, 2018; Nabbout and Thiele, 2020). We have also indicated in the “**Introduction**” that CBD (and other cannabinoids too) may have additional therapeutic properties of interest for the different comorbidities occurring in these syndromes, given its anti-inflammatory, antioxidant and, in general, cytoprotective properties, all of them well-demonstrated during years in adult individuals (Hill et al., 2012; Fernández-Ruiz et al., 2013) and also in the pediatric field against neonatal hypoxia-ischemia and other disorders related to neurodevelopment (Sagredo et al., 2018). This potential has already been investigated in a couple of previous studies (Kaplan et al., 2017; Patra et al., 2020), which demonstrated in animal models of DS the efficacy of CBD in preventing premature mortality and in improving some associated comorbidities (anxiety, depression, autism, motor impairment, and memory defects). In this context, we addressed as a third objective in our current study to explore the status of the endocannabinoid signaling in specific CNS structures of Syn-Cre/Scn1a^WT/A1783V^ mice, in particular those areas more related to comorbidities found in these mice. Our hypothesis is that the endocannabinoid signaling may be dysregulated in DS and that CBD (and other cannabinoids) may be active in correcting such dysregulation, following the data obtained in studies in other neurological disorders (Cristino et al., 2020). In fact, the idea of an endocannabinoid dysregulation in DS was previously investigated in our laboratory in a clinical study consisting in the analysis of lymphocytes isolated from patients (Rubio et al., 2016). We analyzed different components of the endocannabinoid system, intracellular signals down-stream the endocannabinoid-related receptors, and also some potential targets for CBD that are outside the endocannabinoid signaling. Our data revealed a significant increase in CB_2_ receptors, an important element of the endocannabinoid system that has been related to inflammatory responses (Fernández-Ruiz et al., 2007), which was paralleled by increases in some cytokines (e.g., TNF-α, IL-1β), in the PPAR-γ nuclear receptor, and in the marker of lymphocyte activation Cd70 (Rubio et al., 2016), supporting the idea that inflammation could have an important influence in DS pathogenesis and, in general, in drug-resistant infantile epilepsies (Boer et al., 2010; Iyer et al., 2010; Koh, 2018). Similar data (upregulation in the CB_2_ receptor) have been found in the postmortem brain of epileptogenic developmental pathologies (Zurolo et al., 2010) and also in peripheral blood mononuclear cells of children affected by autistic disorders (Siniscalco et al., 2013). Our present study confirmed the relevance of this response, as we found elevated levels of the CB_2_ receptor in the hippocampus (one of the areas in our study, in particular, the dentate gyrus, that was most affected by glial reactivity), although not in other CNS structures, in Syn-Cre/Scn1a^WT/A1783V^ mice. It is possible that this upregulation of the CB_2_ receptor found in the hippocampus means an endogenous protective response aimed at controlling the inflammatory response in DS, as has been found to occur in other neurological disorders (Fernández-Ruiz, 2019), but this would require further research aimed at identifying the neural substrates within the hippocampus where the elevation of CB_2_ receptors take place, as well as to confirm the benefits of compounds targeting this receptor in the control of local inflammatory responses.

The upregulation of the CB_2_ receptor was not the only response of the endocannabinoid system found in Syn-Cre/Scn1a^WT/A1783V^ mice. We also found that the CB_1_ receptor was possibly downregulated in the cerebellum and the hippocampus, supporting the idea of an impairment in the synaptic function played by this receptor by modulating transmitter release in excitatory and inhibitory neurons of both structures, which could be compatible with the motor and memory changes found in these mice. Reductions in the endocannabinoid inactivating enzymes, were also evident in the cerebellum (both MAGL and FAAH), the prefrontal cortex (only MAGL), and the hippocampus (only FAAH) of Syn-Cre/Scn1a^WT/A1783V^ mice, supporting a possible elevation in endocannabinoid levels, in particular 2-AG given that changes in MAGL in Syn-Cre/Scn1a^WT/A1783V^ mice appear to be stronger. Such elevation would need to be confirmed in further studies but may be compatible with the endogenous protective responses exerted by 2-AG in conditions of brain damage, as shown to occur in chronic neurodegenerative diseases (Xu and Chen, 2015) or in brain trauma (Panikashvili et al., 2001).

In conclusion, Syn-Cre/Scn1a^WT/A1783V^ mice exhibited spontaneous convulsions and a greater responsiveness when seizures were induced with PTZ at PND25, accompanied by hyperactivity, certain memory impairment, less anxiety, and an altered social behavior, which represent some important features present in humans. These alterations tended to disappear at days after PND25, with the only exception of altered social interaction, which still persists at PND60, and, in part, lower anxiety. These behavioral impairments were associated with greater glial reactivity and generation of reactive NSCs in the hippocampal neurogenic niche, as well as with changes in some endocannabinoid receptors and enzymes that would support the idea of a possible endocannabinoid dysregulation restricted to specific CNS structures.

## Data Availability Statement

The original contributions presented in the study are included in the article/Supplementary Material, further inquiries can be directed to the corresponding author/s.

## Ethics Statement

The animal study was reviewed and approved by Animal Welfare of the Complutense University (PROEX 033/17).

## Author Contributions

JF-R, OS, and JE contributed to the study design, coordination, and supervision. VS, CA, PD, SM-S, and MR contributed with all animal breeding and genotyping, behavioral testing, sample collections, and/or biochemical and histological analyses. JF-R with OS, VS, and CA contributed to the statistical analysis of the data. JF-R contributed to the manuscript writing with the revision and approval of all authors. All authors read and approved the final manuscript.

## Conflict of Interest

The authors declare that the research was conducted in the absence of any commercial or financial relationships that could be construed as a potential conflict of interest.

## Footnotes

^1^https://www.fda.gov/NewsEvents/Newsroom/PressAnnouncements/ucm611046.htm

^2^https://www.ema.europa.eu/en/medicines/human/EPAR/epidyolex

## Supplementary Material

Supplementary File 1 Representative PCRs corresponding to the genotyping of Scn1a^WT/WT^, Syn-Cre/Scn1a^WT/WT^, Scn1a^WT/A1783V^, and Syn-Cre/Scn1a^WT/A1783V^ mice. In the Scn1a PCR, wild-type alleles corresponded to 461 bp, whereas mutant alleles corresponded to 496 bp. In the Cre PCR, the band corresponded to 300 bp.

Supplementary File 2 Video recording of spontaneous seizures shown by one Syn-Cre/Scn1a^WT/A1783V^ mouse.

Supplementary File 3 Comparison of the immunoreactivity for the astroglial marker GFAP and the microglial marker Iba-1, measured by immunofluorescence in the prefrontal cortex and the hippocampal dentate gyrus, among the three control groups (Scn1a^WT/WT^, Syn-Cre/Scn1a^WT/WT^, and Scn1a^WT/A1783V^ mice) for Syn-Cre/Scn1a^WT/A1783V^ mice at the PND25. Values are means ± SEM of more than six animals per group. Data were assessed by using one-way ANOVA followed by the Bonferroni test (GFAP: prefrontal cortex: *F*_(2, 17)_ = 1.458, ns; hippocampal dentate gyrus: *F*_(2, 17)_ = 1.094, ns; Iba-1: prefrontal cortex: *F*_(2, 17)_ = 0.076, ns; hippocampal dentate gyrus: *F*_(2 17)_ = 0.203, ns).

Supplementary File 4 Representative blots corresponding to the analysis of protein levels for the CB_1_ and CB_2_ receptors and the FAAH and MAGL enzymes in the prefrontal cortex and the hippocampus of Syn-Cre/Scn1a^WT/A1783V^ mice compared with Scn1a^WT/W^ animals at PND25. As indicated in the “Materials and Methods” section, images were analyzed with Image Lab software (Bio-Rad Laboratories, CA, USA) to generate the quantitative data presented in Figure 11, which corresponded to mean ± SEM of values of the ratio between the optical densities of the specific protein (CB_1_, CB_2_, FAAH, MAGL) band and the total protein (escalated according to molecular weights) measured in membranes for each sample. As indicated in the legend to Figure 11, the two experimental groups consisted of five to six different individual data each coming from a different animal.
