# Supplementary figures and images for: Neuropathological Characterization of a Dravet Syndrome Knock-In Mouse Model Useful for Investigating Cannabinoid Treatments
Source: Front Mol Neurosci. 2021 Jan 29;13:602801. doi: 10.3389/fnmol.2020.602801 (PMC7879984; doi:10.3389/fnmol.2020.602801)

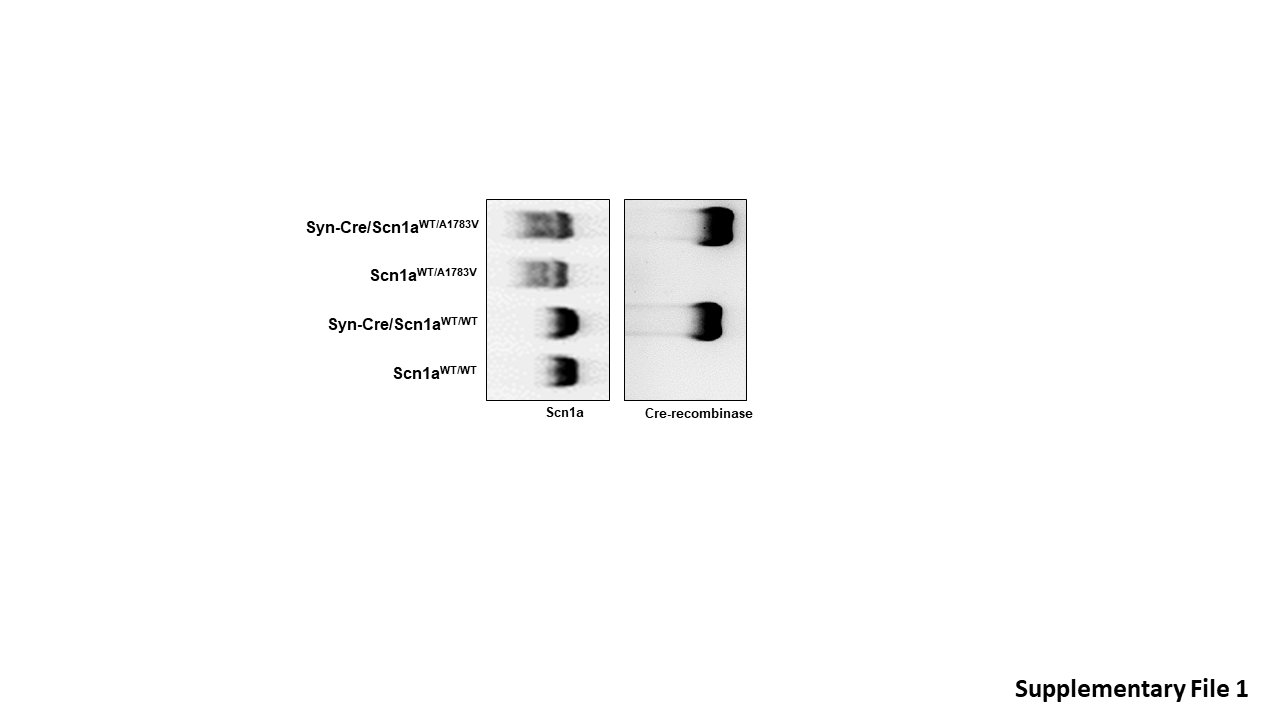

Supplement: Supplementary File 1 — Representative PCRs corresponding to the genotyping of Scn1aWT/WT, Syn-Cre/Scn1aWT/WT, Scn1aWT/A1783V, and Syn-Cre/Scn1aWT/A1783V mice. In the Scn1a PCR, wild-type alleles corresponded to 461 bp, whereas mutant alleles corresponded to 496 bp. In the Cre PCR, the band corresponded to 300 bp. [file Image_1.TIF]

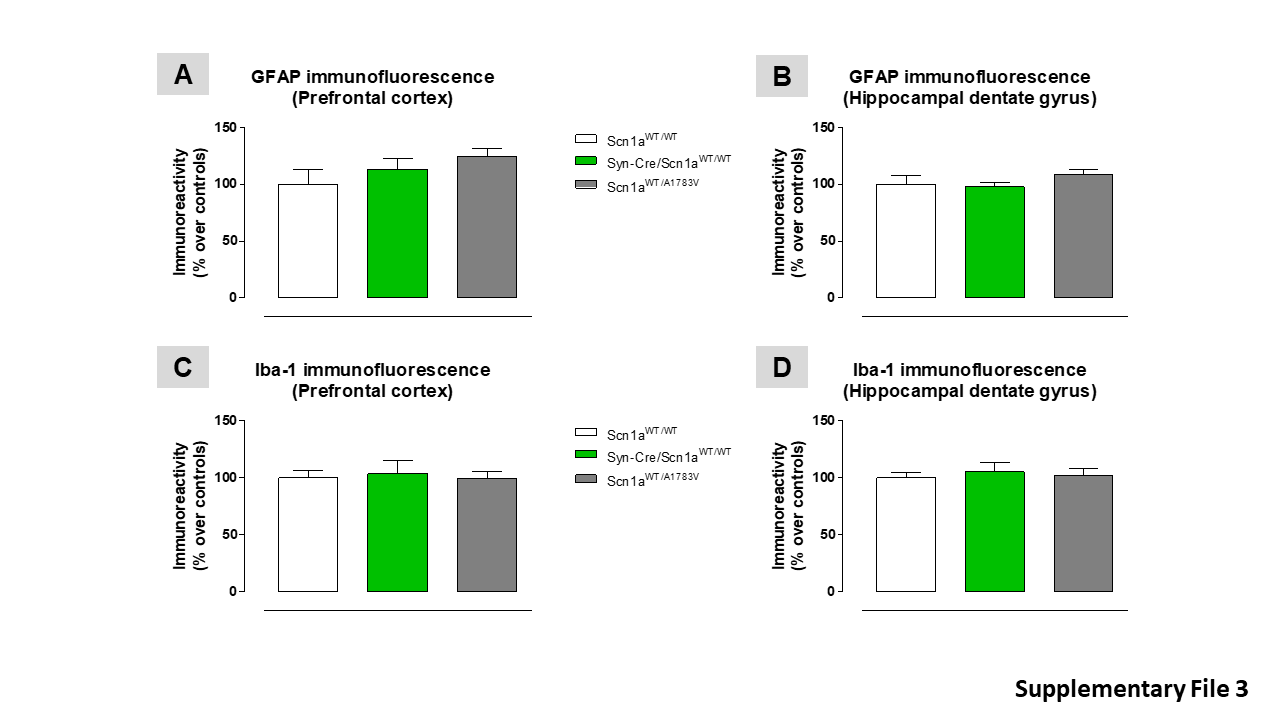

Supplement: Supplementary File 3 — Comparison of the immunoreactivity for the astroglial marker GFAP and the microglial marker Iba-1, measured by immunofluorescence in the prefrontal cortex and the hippocampal dentate gyrus, among the three control groups (Scn1aWT/WT, Syn-Cre/Scn1aWT/WT, and Scn1aWT/A1783V mice) for Syn-Cre/Scn1aWT/A1783V mice at the PND25. Values are means ± SEM of more than six animals per group. Data were assessed by using one-way ANOVA followed by the Bonferroni test (GFAP: prefrontal cortex: F(2, 17) = 1.458, ns; hippocampal dentate gyrus: F(2, 17) = 1.094, ns; Iba-1: prefrontal cortex: F(2, 17) = 0.076, ns; hippocampal dentate gyrus: F(2 17) = 0.203, ns). [file Image_3.tif]

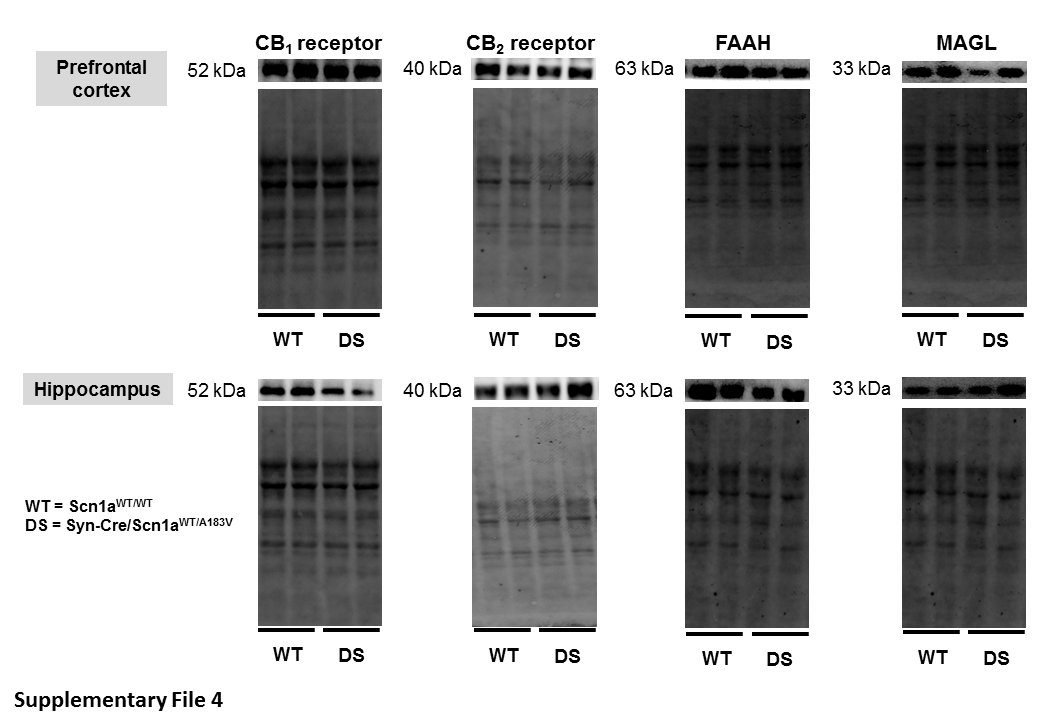

Supplement: Supplementary File 4 — Representative blots corresponding to the analysis of protein levels for the CB1 and CB2 receptors and the FAAH and MAGL enzymes in the prefrontal cortex and the hippocampus of Syn-Cre/Scn1aWT/A1783V mice compared with Scn1aWT/W animals at PND25. As indicated in the “Materials and Methods” section, images were analyzed with Image Lab software (Bio-Rad Laboratories, CA, USA) to generate the quantitative data presented in Figure 11, which corresponded to mean ± SEM of values of the ratio between the optical densities of the specific protein (CB1, CB2, FAAH, MAGL) band and the total protein (escalated according to molecular weights) measured in membranes for each sample. As indicated in the legend to Figure 11, the two experimental groups consisted of five to six different individual data each coming from a different animal. [file Image_4.tif]
